# Mosaic human preimplantation embryos and their developmental potential in a prospective, non-selection clinical trial

**DOI:** 10.1016/j.ajhg.2021.11.002

**Published:** 2021-11-18

**Authors:** Antonio Capalbo, Maurizio Poli, Laura Rienzi, Laura Girardi, Cristina Patassini, Marco Fabiani, Danilo Cimadomo, Francesca Benini, Alessio Farcomeni, Juliana Cuzzi, Carmen Rubio, Elena Albani, Laura Sacchi, Alberto Vaiarelli, Matteo Figliuzzi, Necati Findikli, Onder Coban, Fazilet K. Boynukalin, Ivan Vogel, Eva Hoffmann, Claudia Livi, Paolo E. Levi-Setti, Filippo M. Ubaldi, Carlos Simón

**Affiliations:** 1 Igenomix, Reproductive Genetics, Marostica 36063, Italy; 2 Clinica Valle Giulia, GeneraLife IVF, Rome 00197, Italy; 3 DEMETRA, GeneraLife IVF, Florence 50141, Italy; 4 Department of Economics and Finance, University of Rome “Tor Vergata,” Rome 00133, Italy; 5 Igenomix US & Canada, Miami, FL 33126, USA; 6 Igenomix, Valencia 46980, Spain; 7 Igenomix Foundation, Reproductive Genetics, Valencia 46980, Spain; 8 Istituto di Ricovero e Cura a Carattere Scientifico, Humanitas Research Hospital, Division of Gynecology and Reproductive Medicine, Fertility Center, Rozzano (Milan) 20089, Italy; 9 Bahceci Fulya IVF Centre, Embryology Laboratory, Istanbul 34394, Turkey; 10 Department of Biomedical Engineering, Beykent University, Istanbul 34398, Turkey; 11 British Cyprus IVF Hospital, Nicosia 2681, Cyprus; 12 Bahceci Fulya IVF Centre, Infertility Clinic, Istanbul 34394, Turkey; 13 Danish National Research Foundation Center for Chromosome Stability, Department of Cellular and Molecular Medicine, Faculty of Health and Medical Sciences, University of Copenhagen, Copenhagen 2200, Denmark; 14 Valencia University and INCLIVA, Department of Obstetrics and Gynecology, Valencia, 46010, Spain; 15 School of Medicine, Department of Obstetrics and Gynecology, Harvard University, Cambridge, MA 02115, USA

## Abstract

Chromosome imbalance (aneuploidy) is the major cause of pregnancy loss and congenital disorders in humans. Analyses of small biopsies from human embryos suggest that aneuploidy commonly originates during early divisions, resulting in mosaicism. However, the developmental potential of mosaic embryos remains unclear. We followed the distribution of aneuploid chromosomes across 73 unselected preimplantation embryos and 365 biopsies, sampled from four multifocal trophectoderm (TE) samples and the inner cell mass (ICM). When mosaicism impacted fewer than 50% of cells in one TE biopsy (low-medium mosaicism), only 1% of aneuploidies affected other portions of the embryo. A double-blinded prospective non-selection trial (NCT03673592) showed equivalent live-birth rates and miscarriage rates across 484 euploid, 282 low-grade mosaic, and 131 medium-grade mosaic embryos. No instances of mosaicism or uniparental disomy were detected in the ensuing pregnancies or newborns, and obstetrical and neonatal outcomes were similar between the study groups. Thus, low-medium mosaicism in the trophectoderm mostly arises after TE and ICM differentiation, and such embryos have equivalent developmental potential as fully euploid ones.

## Introduction

Aneuploidy in human conceptions is the leading cause of embryo implantation failure, pregnancy loss, and congenital disorders in live-born infants. Although chromosome segregation errors in oocytes are its major cause,^1^ recent observations suggest that aneuploidy can also arise significantly during preimplantation embryonic divisions.^2^ Aneuploidies due to meiotic errors are present uniformly throughout the embryo, whereas those with a mitotic origin (i.e., post-fertilization) give rise to cell lineages with different chromosomal content (embryonic mosaicism). Understanding the incidence of mosaicism and the distribution of aneuploid cells throughout the embryo is important because genetic testing of preimplantation embryos and prenatal fetuses is carried out, respectively, on a trophectoderm biopsy or samples of placental tissue originated from it, rather than the cell lineage programmed to form the embryo proper (i.e., inner cell mass (ICM), ICM).

**Figure 1:**
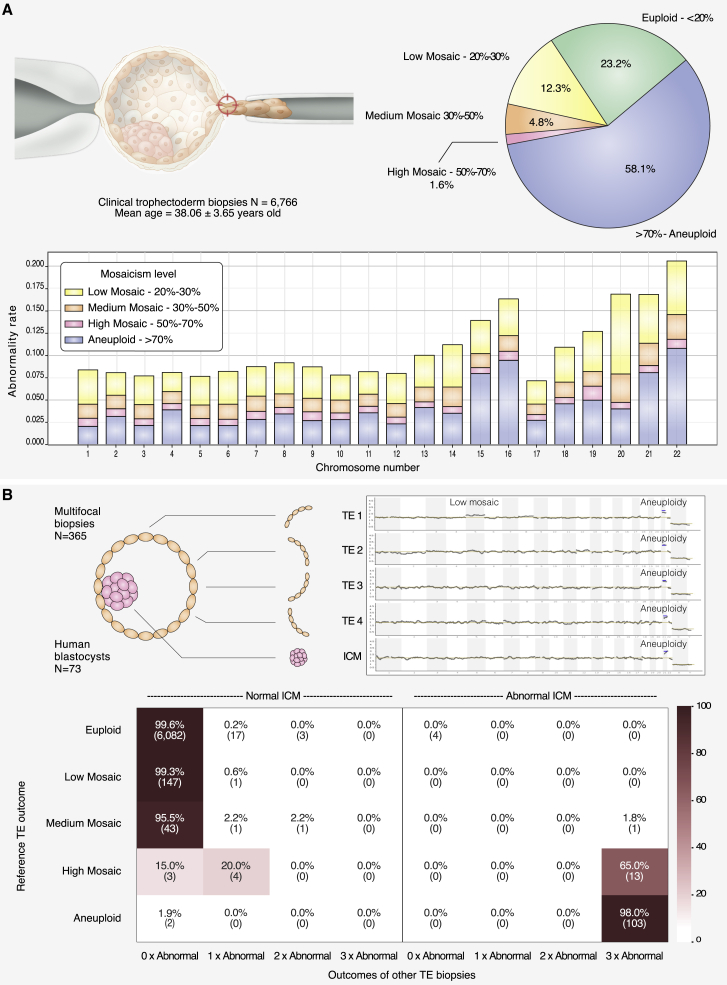
Aneuploidy incidence in clinical trophectoderm biopsies and embryonic concordance study (A) The pie chart represents the distribution of aneuploidy categories observed in 6,766 clinical trophectoderm (TE) biopsies analyzed in our PGT-A diagnostic setting (classified according to the most severe abnormality across all chromosomes). The stacked bar chart represents the incidence of each aneuploidy category at the chromosome level. (B) Top left: 73 human blastocysts were disaggregated into five portions: four TE biopsies and the inner cell mass (ICM ) biopsy. Top right: examples of PGT-A plots displaying a low mosaic configuration confined to TE1 and a uniform aneuploidy detected in all portions. Bottom: this heatmap shows diagnostic concordance rates per chromosome on the basis of 73 embryos with five biopsies each (365 embryo biopsies), leading to 6,424 comparisons (73 embryos × 22 autosomal chromosomes × 4 permutations of reference biopsy). One of the four TE biopsies is considered as a reference, whereas the remaining biopsies (three TE + ICM) are used for verifying the outcome of the reference. Based on its copy-number result, each autosomal chromosome within the reference biopsy is classified into one of five categories (euploid, low-grade mosaic [20–30%], medium-grade mosaic [30–50%], high-grade mosaic [50–70%], or aneuploid; rows), whereas the verification biopsies are classified in two categories (normal [<50%] or abnormal [>50%]). The heatmap shows concordance rates between the reference TE and the three verification TE biopsies (columns), given the outcome of the reference (rows). This analysis is split into two maps depending on whether the ICM is normal (left) or abnormal (right). For instance, the cell in the first column, second row, indicates that if a chromosome is detected at a low-grade mosaic configuration in the reference TE biopsy, there is a 99.3% probability that a normal diagnostic outcome is detected in all four other verification biopsies. The risk of chromosomal abnormalities in the four remaining embryonic portions is rare and similar when the reference biopsy shows a euploid, low-grade mosaic, or medium-grade mosaic outcome.

With the introduction of high-resolution next-generation sequencing (NGS) protocols, chromosomal mosaicism has been reported in up to 20% of clinical trophectoderm (TE) biopsies^3^ (Figure 1A), which consist of a sample of 3 to 10 cells. Recent large genome-wide non-invasive prenatal screening (NIPS) studies performed at 12 weeks of gestation have shown that the presence of confined placental mosaicism (CPM) explains a significant fraction of false-positive cases of rare autosomal trisomies (RATs).^4^^,^^5^ Yet, the prevalence of chromosomal mosaicism in human pregnancies is reported in fewer than 0.3% of prenatal tests (e.g., by amniocentesis or by NIPS).^6^ On the basis of experiments in murine models, the sharp drop in mosaicism between pre- and post-implantation stages has been explained by the selective elimination of aneuploid cells through competitive growth of euploid cells or apoptosis of the abnormal cellular clones.^7^^,^^8^ In humans, the prevalence and developmental potential of mosaic embryos consisting of both diploid and aneuploid cells remain the subject of intense debate.^9^ This is largely due to a paucity of studies that have assessed mosaicism across the whole embryo; as a result, our understanding of the distribution of aneuploid cells is limited. One major clinical impact of this shortcoming has been that fewer than 3% of mosaic embryos are being used in in babies born after in vitro fertilization (IVF) treatment^10^, ^11^, ^12^ as a result of concerns that the aneuploidy detected in the TE might affect the ICM and, therefore, potentially the baby. However, mosaicism is not generally detected at increased rates IVF treatment,^13^, ^14^, ^15^ suggesting that aneuploid cells in mosaic preimplantation embryos do not contribute toward the genetic make-up of live-born infants. Consistent with this hypothesis, a small study of mosaic embryos showed they are capable of giving rise to healthy live births.^16^

Larger, retrospective studies have concluded that mosaic embryos have a lowered reproductive potential.^10^^,^^17^ Such retrospective data, however, are affected by a strong selection bias. In particular, retrospective analyses do not take into account the fact that mosaic embryos are transferred as a last option and, consequently, that their reproductive performance is often measured on a highly selected subpopulation of women who had previous failed implantations with euploid embryos. Mosaic embryos are also transferred in those individuals producing only aneuploid embryos, introducing again a strong selection bias toward a poor-prognosis population. For example, in the largest retrospective study published to date where the reproductive competence of putative mosaic embryos was assessed, 94.6% of cases involving mosaic-embryo transfer were included because no uniformly euploid embryos were available.^17^ Accordingly, the reproductive potential of mosaic embryos remains to be tested in a robust, well-powered prospective, non-selection clinical trial.

Here, we have first investigated the prevalence and distribution of aneuploid cells in the largest dataset of disaggregated human blastocysts currently available. Our findings show that in the majority of cases where mosaicism is detected in TE biopsies, this is due to a few aneuploid cells that originate from the trophectoderm tissue and give rise to a low- to medium-grade mosaicism configuration. To provide exhaustive evidence on the effects that highly localized aneuploid cells have on a mosaic embryo’s clinical performance, we carried out a prospective non-selection study allowing an unbiased comparison between uniformly euploid and mosaic embryos in terms of reproductive potential and chromosomal normalcy between uniformly euploid and mosaic embryos, providing exhaustive evidence related to their clinical performance and the ensuing offspring. We report that putative mosaic embryos show clinical outcomes similar to those of uniformly euploid embryos and that there are not any significant implications for pregnancy and live-birth outcomes or the offspring’s health. The clinical data generated from this trial will clearly resolve major concerns related to the management of mosaicism findings in preimplantation genetic testing for aneuploidies (PGT-A) and will be of fundamental importance for helping many infertile individuals to make more informed reproductive decisions.

## Materials and methods

### Assessment of mosaicism incidence and prevalence in human blastocysts

Data from clinical cases, consisting of 6,766 clinical TE biopsies performed in 2020 and 2021, were recovered so that the incidence of different aneuploidy categories observed in the diagnostic setting of PGT-A could be computed. A total of 91 blastocyst-stage human embryos were donated to research at the British Cyprus IVF Hospital under ethical-committee approval obtained from the institutional review board at Near East University (project number YDU/2018/64-685). Approved informed-consent forms were signed by all the individuals donating their embryos to this study. Embryos were warmed, and ICM biopsies were isolated by a previously validated mechanical method.^18^ The TE was then disaggregated in four equally sized portions. Blinded NGS analysis was performed on all re-biopsies, and results were analyzed so that the prevalence and distribution of abnormal cells in the remaining embryonic sections could be assessed according to the original estimated mosaicism rate in the reference TE biopsy (see supplemental methods).

### Clinical trial design and participants

We conducted a multicenter, double-blinded, non-selection trial (trial registration NCT03673592) involving consecutive IVF with blastocyst-stage PGT-A treatments followed by frozen euploid, low-grade, or medium-grade mosaic single-embryo transfer (SET). Women eligible for participation were below the age of 45, had autologous oocytes, were undergoing intra-cytoplasmic sperm injection (ICSI) for all oocytes, and had at least one transferrable embryo available (euploid or low- to moderate-grade mosaic). Treatment cycles were excluded from the study if the embryo to be transferred showed the worst morphological grade according to an adaptation of Gardner’s criteria^19^ or if the female partner had a chronic medical condition associated with adverse pregnancy outcomes. The purpose this criterion was to mitigate an intrinsic bias; euploid blastocysts of very poor morphological grade were shown to result in lower live-birth and higher miscarriage rates than embryos with better morphology.^20^ IVF procedures were carried out according to standard practices employed at each clinic (supplemental methods).

The trial was conducted in compliance with the International Conference on Harmonisation and the Declaration of Helsinki. The protocol was approved by the institutional review board of Clinica Valle Giulia, Rome (September 3, 2018) and the Humanitas Research Hospital Ethics Committee, Rozzano (code 477/19). First participants were recruited beginning September 20, 2018. All the individuals eligible for the study provided written informed consent before starting ovarian stimulation. Inclusion in the study population was validated at the time of transfer, when all acceptance criteria were confirmed (i.e., availability of non-aneuploid embryos). The trial was registered with ClinicalTrials.gov as NCT03673592.

### Intervention

After TE biopsy and NGS-based chromosomal analysis, a diagnostic report on embryos’ chromosomal status was sent to the clinical sites (supplemental methods). Embryos showing a low or moderate degree of chromosomal mosaicism were blindly reported as euploid without distinction from uniformly euploid embryos. Among those reported as euploid, embryos were selected for SET on the basis of standard morphological features, providing a blinded allocation of the participants into the three main categories “euploid” (group A), “low-grade mosaic” (group B; 20–30% aneuploid cells), and “medium-grade mosaic” (group C; 30–50% aneuploid cells). Cases were followed up on during the post-transfer, gestational, and postnatal periods. The chromosomal status of 38 newborns derived from the transfer of putative mosaic embryos was investigated via single-nucleotide polymorphism arrays (SNPa genotyping) on saliva samples collected from the newborns and their parents. Genotyping data of the trios were used for investigating any potential instance of mosaicism or uniparental disomies (UPDs) in the offspring. Details of the genotyping protocol are reported in the supplemental methods.

### Outcomes

The primary outcome measure was sustained implantation rate (the probability that any transferred embryo will implant and progress to delivery),^21^ defined as live-birth rate (LBR) per transferred embryo according to the World Health Organization and International Committee for Monitoring Assisted Reproductive Technologies International Glossary on Infertility and Fertility Care.^22^ The LBR was calculated as the number of newborns delivered on or after 22 weeks of gestation over the number of embryos replaced. In the event of a SET, as occurred in this study for all cases, the metric is identical to delivery rate per transfer. The secondary outcome was miscarriage rate, defined as the spontaneous loss of an intra-uterine pregnancy prior to 20 completed weeks of gestational age. This included the evaluation of pregnancy rate (PR) and biochemical pregnancy (BP). Mean gestational age at birth and birth weight were collected as neonatal outcomes. Adverse outcomes were determined by the detection of chromosomal abnormalities, including uniparental disomy, in the miscarried product of conception (POC) during prenatal diagnosis (PND; amniocentesis/chorionic villi sampling CVS) and/or at birth.

The implication of excluding putative mosaic embryos from clinical use has been evaluated in consideration of the potential loss of live births in a given IVF treatment cycle (cumulative LBR, CLBR per cycle) by two different methods: (1) using actual data from embryo transfer in the study period but excluding live births achieved from low- and moderate-mosaic embryos and (2) by modeling the optimistic scenario where all transferable embryos are replaced. A complete description of the outcome of these analyses is reported in the supplemental methods.

### Statistical analysis

The primary endpoint for this analysis was the non-inferiority of LBR when euploid and mosaic embryos were compared. Assuming an LBR of 45% for uniformly euploid embryos versus 42.5% for low- or moderate-degree mosaics,^2^ a 1:1 sampling ratio for the two groups, and a planning non-inferiority margin of 7.5%,^23^^,^^24^ we calculated that 421 embryos per group would guarantee the power of at least 90% for a significance level set at 5%. The same sample size was sufficient for targeting non-inferiority claims in the miscarriage rate. Assuming a 10% miscarriage rate for uniformly euploid embryos and 15% for moderate to low mosaics, and setting a significance level at 5%, we also found the sample size had >90% power to assess non-inferiority in the miscarriage rate between control and test groups; the non-inferiority margin was 2%.

Data are expressed as mean ± standard deviation or percentages as appropriate. Proportions were compared via the chi-square test or Fisher exact test for 2 × 2 contingency tables. The non-inferiority endpoint was set as the 95% CI for difference in proportions lying below the planned margin. In addition to computing confidence intervals and p values for the difference in proportions, we computed the odds ratios (ORs) and adjusted odds ratios (AORs) of mosaicism for LBR, PR, IR, MC, and BP through logistic regression models. In multivariate analyses, ORs were adjusted for female age, male age, center, morphology of the blastocyst, day of the biopsy, number of previous implantation failures, previous miscarriages, previous live birth, infertility indication, and sperm origin (ejaculated versus surgical). All other tests were two tailed. Data had no missing values for variables involved in the primary analysis. A complete case approach was used for secondary analyses. All analyses were conducted with SPSS v. 21 and R v. 3.5.1.

## Results

### Incidence and prevalence of chromosomal mosaicism on blastocyst-stage human embryos

To shed light on the incidence and prevalence of chromosomal mosaicism in human preimplantation embryos, we first analyzed a large historical dataset of 6,766 embryos, where a clinical trophectoderm (cTE) biopsy had been processed with NGS technology. This approach could detect a mosaicism rate as low as 20% in cell line mixture models.^25^ This clinical dataset of cTE biopsies (n = 6,766; mean maternal age n = 38.06; SD 3.65) revealed an incidence of diploid-aneuploid mosaicism of 18.7%; smaller chromosomes were more frequently represented than others (Figure 1A).

Next, we investigated the prevalence of mosaicism and its clonal distribution in 91 unselected human blastocysts that were donated for research. We divided the trophectoderm into four biopsies and isolated the ICM by using a validated mechanical method able to retrieve ICM specimens free of TE contamination (Figure 1B).^18^ The collection of all five samples from the same blastocyst was successful for 75 out of 91 embryos. Blinded NGS analysis generated an informative result for all five samples in 73 embryos, allowing robust diagnosis for each of the four TE biopsies and the ICM. Chromosomal mosaicism rates were calculated for each of the five blastocyst biopsies, and the concordance rate between the reference TE biopsy and the remaining four embryonic portions (including the ICM) was assessed on the basis of permutation analysis (Figure 1B). Using one TE biopsy as a reference, we found that chromosomal abnormalities in the ICM were extremely rare and not statistically different irrespective of whether the reference biopsy had been classified as euploid or low- to medium-grade mosaic (p = 0.14). In these cases, segregation patterns were confined to a single portion of the TE and rarely affected the ICM **(**Figure 1B). In contrast, high-grade mosaicism (50–70%) in a single TE sample was commonly associated with uniform aneuploidy throughout the embryo (including the ICM) in 65% of cases (13 out of 20 embryos; 95% CI = 43–82%; Figure 1B). We conclude that, in the vast majority of cases, the detection of low- to medium-grade mosaicism in one TE biopsy reflects a status of highly confined aneuploidy rather than its random distribution throughout the embryo.

### Prospective non-selection clinical-trial results

**Figure 2:**
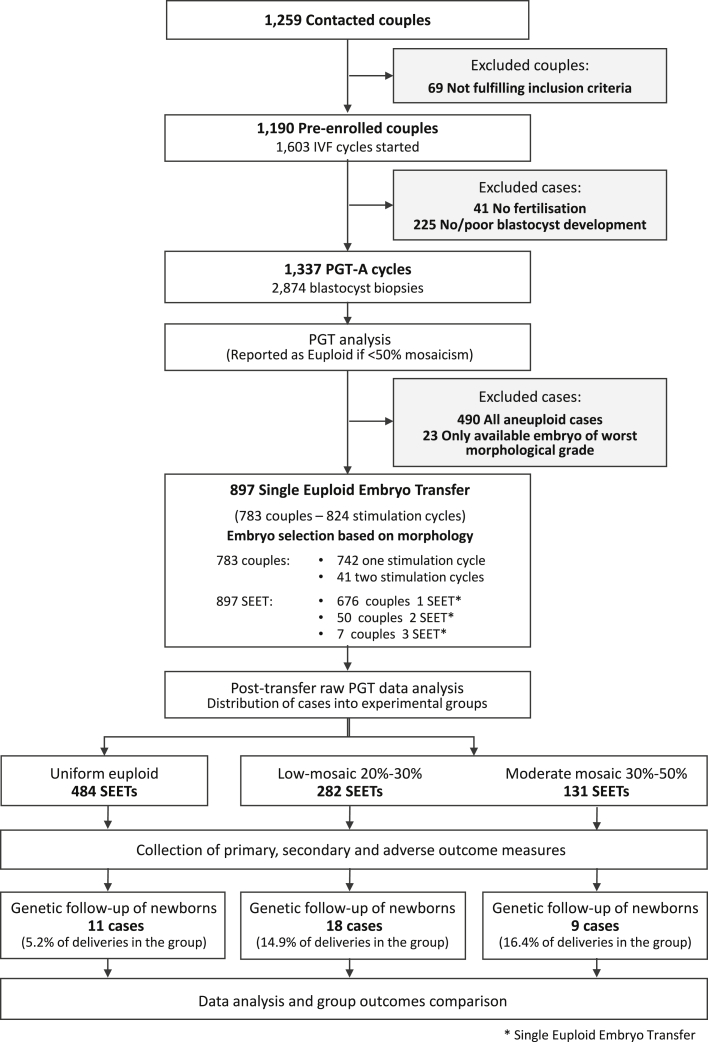
Study flow chart

Our data suggest that low- to medium-grade mosaic embryos might have developmental potential similar to that of euploid ones. So far, clinical outcomes of mosaic embryos have only been compared retrospectively in selected subpopulations of individuals who failed to get pregnant with previous transfers of euploid embryos or who only had one putative mosaic embryo available and were therefore of poor prognosis.^7^^,^^10^, ^11^, ^12^ To produce unbiased evidence of the reproductive potential of low- to medium-grade mosaic embryos, we carried out a multicenter double-blinded non-selection clinical trial involving 1,190 couples and 1,603 IVF cycles (trial registration number NCT03673592). All cycles included blastocyst-stage preimplantation genetic testing for aneuploidy (a single clinical TE biopsy) and were initiated on September 20^th^, 2018 and ended on December 31^st^, 2019 (Figure 2). Embryos showing low- and medium-grade chromosomal mosaicism were blindly reported (non-selection) as euploid and transferred via SET based on standard morphological features.^19^ Embryos were blindly allocated to three main categories: euploid (group A), low-grade mosaic (group B) (20–30% aneuploid cells), and medium-grade mosaic (group C) (30–50% aneuploid cells). The primary outcome measure was sustained implantation rate through delivery (presence of a viable pregnancy after 20 weeks of gestation), measured as the live birth rate (LBR), defined by the WHO as live births per embryos transferred. The secondary outcome measure was miscarriage rate, defined by loss of an intra-uterine pregnancy prior to the 20 completed weeks of gestational age. The distribution of mosaic chromosome types across each group of transferred mosaic embryos is reported in Figure S1**.** Baseline characteristics of study participants and main effects of treatments on those participants' IVF cycles were similar among the three groups and are shown in Table S1.

We found no evidence that low- or medium-grade mosaicism affected live birth rates among 484 uniform euploid embryos (group A), 282 low-degree mosaic embryos (group B), or 131 medium-degree mosaic embryo transfers (group C) included in the primary analysis, which was powered for the LBR as a primary outcome. LBRs of uniformly euploid embryos and low- and moderate-grade mosaic embryos were 43.4% (95% CI = 38.9–47.9%), 42.9% (95% CI = 37.1–48.9%), and 42% (95% CI = 33.4–50.9%), respectively (Table 1). The fact that the confidence interval for the difference fell below the planned 7.5% margin (95% CI = −5.7–7.3%) shows that the non-inferiority endpoint for the primary outcome measure was met, suggesting similar reproductive outcomes for euploid and low- and moderate-grade mosaic embryos. No difference was observed in miscarriage rates (OR = 0.89; 95% CI = 0.50–1.55; p = 0.69), providing additional support for chromosomal normalcy of pregnancies from low- and medium-grade mosaic embryos (Table 1). Additionally, the number of chromosomes showing a mosaic configuration (commonly referred as complex mosaic) was also not associated with any of the outcomes investigated (see Table S2). At a multivariate-analysis level, an effect on LBR was observed for poor-quality blastocyst morphology (adjusted odds ratio (AOR) = 0.56 compared to the top-quality category, 95% CI = 0.35-0.89; p = 0.0146), day of biopsy (e.g., day 5 versus day 6) (AOR = 0.68 per day, 95% CI = 0.51–0.90, p = 0.008), and surgical origin of sperm (AOR = 0.158, 95% CI = 0.04-0.75, p = 0.020).

**Table 1:** Reproductive outcomes of euploid and mosaic embryos

	Group A: Euploid	Group B: Low-grade mosaic (20–30% variation)	Group C: Medium-grade mosaic (30–50% variation)	Adjusted OR (95% CI; p value)
Test sets, n	484	282	131	-
Positive pregnancy test, % (n)	55.8% (270/484)	55.0% (155/282)	55.7% (73/131)	0.98 (0.75–1.27; 0.86)
Biochemical pregnancy loss, % (n)	10.7% (29/270)	12.3% (19/155)	13.7% (10/73)	1.18 (0.69–2.02; 0.53)
Miscarriage, % (n)	12.0% (29/241)	11.0% (15/136)	12.7% (8/63)	0.89 (0.50–1.55; 0.69)
Live birth, % (n)	43.4% (210/484)	42.9% (121/282)	42.0% (55/131)	0.97 (0.74–1.26; 0.82)
Monochorial twins delivery, n	1	1	1	-
Gestational age, mean (95% CI)	38.4 (38.0–38.7)	38.2 (37.9–38.6)	38.1 (38.0–38.5)	-
Birth weight, mean (95% CI)	3,286 (3,200–3,371)	3,174 (3,080–3,267)	3,130 (2,950–3,310)	-

Biochemical pregnancy is defined by a positive pregnancy test. Implantation rate is defined as the number of gestational sacs observed by vaginal ultrasound at the fifth gestational week divided by the number of embryos transferred. Multiple pregnancy is defined by any scan with more than one heartbeat or gestational sac at the stage of clinical pregnancy (approximately 6 weeks). Miscarriage is defined as the loss of a clinical pregnancy, excluding ectopic pregnancies, before 20 weeks of gestation. A live birth is defined as a delivery that resulted in at least one live birth after 22 weeks of gestation. CI denotes confidence interval. “-” indicates not applicable.

Although our study was not powered to detect mosaicism in pregnancies, we nevertheless investigated potential mosaicism by conducting follow-up analysis of all the available products of conceptions (POCs) after spontaneous miscarriages and elective prenatal diagnosis procedures. Miscarriage rates were similar in the three groups (Table 1). Four of the 52 miscarriage cases were analyzed by standard cytogenetics, all of which were euploid. Twenty-six sustained pregnancies (26 out of 388, 6.7%) underwent prenatal diagnosis by amniocentesis (group A = 15; group B = 6; group C = 3) or by chorionic villi sampling (group A = 2). These numbers were small because the reduction in the use of invasive prenatal diagnosis is one of the aims of PGT-A. All prenatal diagnoses showed euploid karyotype except in one pregnancy from group A (uniformly euploid), where confined placental mosaicism for trisomy 22 (20% abnormal cells) was detected at chorionic villi sampling analysis. However, subsequent follow-up amniocentesis displayed a normal euploid cytogenetic result.

**Figure 3:**
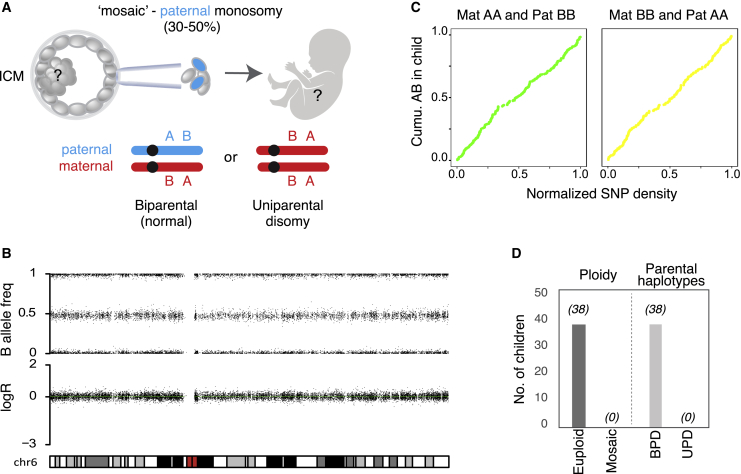
Euploid biparental inheritance in children born from “mosaic” embryo transfer (A) Illustration of a mosaic paternal monosomy inferred from the trophectoderm biopsy. The fetal tissues derive from the inner cell mass, which might contain biparental or uniparental chromosomes or a mixture of them. Supporting SNPs where the maternal and paternal genotypes are homozygous but carry opposite alleles (AA and BB or vice versa) can be used for determining the presence or absence of parental chromosomes. (B) LogR and B allele frequencies for chromosome 6 from a child born from group C. (C) Cumulative AB genotypes in the child of supporting SNPs across chromosome 6. (D) Number (No.) of children investigated with post natal SNPa testing. Total number of samples showing euploid or mosaic karyotype (“ploidy”) or containing both parental chromosomes (biparental disomy, BPD) or two homologous chromosomes from the same parent (UPD).

At birth, obstetric and neonatal outcomes were similar between mosaic and euploid embryos (Table 1). One developmental abnormality was observed among the moderate-grade mosaicism cases (group C), where a baby was born with a diagnosis of Beckwith-Wiedemann Syndrome caused by hypomethylation in the region KvDMR/IC2.^26^ This condition is not a diagnostic target of PGT-A nor due to chromosomal mosaicism. To further investigate any potential persistence of the abnormal cell line after mosaic embryo transfer, we carried out postnatal genotyping of newborns on a subset of 38 families willing to participate (9.8% of all newborns derived from the study; Figure 3). At genome-wide resolution, all genotyping tests showed fully normal karyotypes and the absence of uniparental ditype configuration in the cohort of babies born from mosaic-embryo transfer (Figure 3 and Figure S2).

To determine the clinical impact of not transferring low- and medium-grade mosaic embryos, we developed a theoretical model of cumulative treatment outcomes on the basis of the incidence of mosaicism and clinical outcomes from this trial (∼43% LBR). If we model a scenario that considers the embryos transferred in the trial, an overall reduction of 24 and 7% in LBR would be expected if low- and medium-grade mosaic embryos or if medium-grade embryos only were removed, respectively (Figure S3A). If we consider an optimistic scenario that assumes all potentially available embryos are transferred for a given IVF treatment cycle, not transferring group B and C or group C alone would have resulted in an overall reduction in cumulative LBR of 36 and 11%, respectively (Figure S3B; source data from Tables S3 and S4).

## Discussion

Since the introduction of NGS in preimplantation genetic testing for aneuploidy (PGT-A), there has been substantial debate over the incidence and the distribution of aneuploid cells throughout human preimplantation embryos and how this might affect developmental potential. In this study, we combined the assessment of both the distribution of aneuploid cells throughout human blastocysts and their impact on the embryo’s reproductive potential. The latter parameter was investigated in a prospective multicenter, double-blind, non-selection trial aimed at assessing the clinical performance and safety of low- and medium-degree mosaic embryos by minimizing biases deriving from skewed populations’ prognoses and embryo-prioritization strategies.

First, we assessed the distribution of aneuploid cells throughout blastocyst embryos by analyzing aneuploidy in 365 biopsies derived from 73 unselected blastocyst embryos. Our data support a model in which aneuploidy is confined to a small cluster of cells in the peripheral placental lineages (i.e., TE) at the peri- and early post-implantation stages. This results in low- and medium-grade mosaic embryos when a single cTE biopsy is taken and the signal from aneuploid cells is “averaged” over 3–10 cells. In effect, our disaggregation experiments suggest that once the aneuploid cells have been removed, the remaining embryo consists of euploid cells.

The subsequent prospective, non-selection clinical trial found no evidence of inferior performance of low- and medium-grade mosaic embryos with regards to pregnancy outcomes, including LBR, pregnancy loss, or chromosomal abnormalities in the pregnancy and in children (Table 1, Figure 3). In fact,^16^ on the basis of the transfer of thousands of mosaic embryos carried out to date, the positive predictive value of a mosaic finding in blastocyst embryos by PGT-A has been confirmed in only a single case.^17^^,^^27^ Accordingly, the evidence from our trial does not support the cascade of procedures commonly triggered by the reporting of mosaicism in PGT-A testing cases, including additional genetic counseling sessions, intensified anxiety and distress in treated individuals, higher costs, increased adoption of invasive prenatal diagnosis,^28^^,^^29^ and its associated risk of iatrogenic abortion (0.3%).

Whereas low- and medium-grade mosaicism do not appear to affect live birth, the outcome of high-grade mosaic embryos is clearly a different matter given that 65% of high-grade mosaic embryos were extensively affected, including in the inner cell mass (Figure 1B). In this study it was impossible to investigate the reproductive outcomes of high-grade mosaic embryos because it would be unethical to do so given the disproportionate impact on women, who would most likely be aggrieved by the transfer of fully aneuploid embryos (e.g., by miscarriage). However, this lack of information is not expected to significantly impact the effectiveness of PGT-A cycles because high-grade mosaicism was detected in only 1.6% of embryos analyzed (Figure 1A). It should be noted that the results reported in this study were obtained through the analysis of raw NGS data independent from any proprietary diagnostic algorithm or chromosome-specific consideration commonly used by PGT-A laboratories. Accordingly, our approach provides all PGT laboratories with common ground that is highly reproducible and independent of specific individual settings. Nevertheless, it is important that each laboratory tests and validates its specific algorithms in prospective non-selection studies like the one presented here. Furthermore, these findings concern whole-chromosome mosaic aneuploidies only and cannot be extended to segmental mosaic configuration, which might follow different trajectories. Using a similar embryo-disaggregation design, we have recently demonstrated that most segmental unbalances detected in human blastocysts are indeed in a mosaic state, in opposition to what is observed for whole-chromosome aneuploidies, which mostly originate after meiotic segregation errors.^1^^,^^30^ Future non-selection studies are needed for the investigation of the clinical predictive values of mosaic segmental abnormalities detected in TE biopsies.

Although it remains unclear whether aneuploid cells arrest, become senescent or apoptotic, or are precursors of placental mosaicism,^31^ our data also support the existence of early developmental bottleneck events able to normalize mosaicism-initiating post-zygotic aneuploidies in human embryos. As shown here, low- and medium-grade mosaic embryos have similar reproductive potential as uniformly euploid embryos and, according to our modeling, deselecting them from clinical treatment^10^ would have resulted in a reduction of LBRs of up to 36% in IVF/PGT-A cycles. Considering that in the US alone, more than 50,000 PGT-A cycles are performed every year, the inclusion of putative mosaic embryos in transfer selection would rescue a remarkable number of embryos and live births. In light of this experience, we encourage the preliminary use of data from non-selection trials before being incorporating new PGT-A algorithms and aneuploidy classification criteria in routine practice.

## Data and code availability

All necessary data are available in the main text or supplemental material. For Figure S3, all data points are provided in the supplemental information. The complete dataset of anonymized clinical records is available upon request.

The code for SNP-array-based UPD and mosaicism analysis of trios is publicly available at the following link: https://github.com/Meiomap/TrioAnalysis.

## Supplemental information

Document S1. Figures S1–S3

Document S2. Article plus supplemental information
