# Supplementary material for: Mosaic human preimplantation embryos and their developmental potential in a prospective, non-selection clinical trial
Source: Am J Hum Genet. 2021 Nov 18;108(12):2238–47. doi: 10.1016/j.ajhg.2021.11.002 (PMC8715143; doi:10.1016/j.ajhg.2021.11.002)
Supplement: Document S1. Figures S1–S3 [file mmc1.pdf]

The American Journal of Human Genetics, Volume *108*

Supplemental information

Mosaic human preimplantation embryos and their developmental potential in a prospective, non-selection clinical trial

Antonio Capalbo, Maurizio Poli, Laura Rienzi, Laura Girardi, Cristina Patassini, Marco Fabiani, Danilo Cimadomo, Francesca Benini, Alessio Farcomeni, Juliana Cuzzi, Carmen Rubio, Elena Albani, Laura Sacchi, Alberto Vaiarelli, Matteo Figliuzzi, Necati Findikli, Onder Coban, Fazilet K. Boynukalin, Ivan Vogel, Eva Hoffmann, Claudia Livi, Paolo E. Levi-Setti, Filippo M. Ubaldi, and Carlos Simón

SUPPLEMENTAL INFORMATION

SUPPLEMENTAL FIGURES

Figure S1 - Chromosome representation of transferred mosaic embryos. Distribution of mosaic chromosomes in embryos from group B (top) and from group C (bottom). Mosaic chromosomes were classified according to mosaicism rate: Low Mosaic Trisomy (20% < mosaicism rate ≤30%), Low Mosaic Monosomy (-30%< mosaic rate ≤-20%), Medium Mosaic Trisomy (30%< mosaic rate ≤50%), Medium Mosaic Monosomy (-50%< mosaic rate ≤-30%).

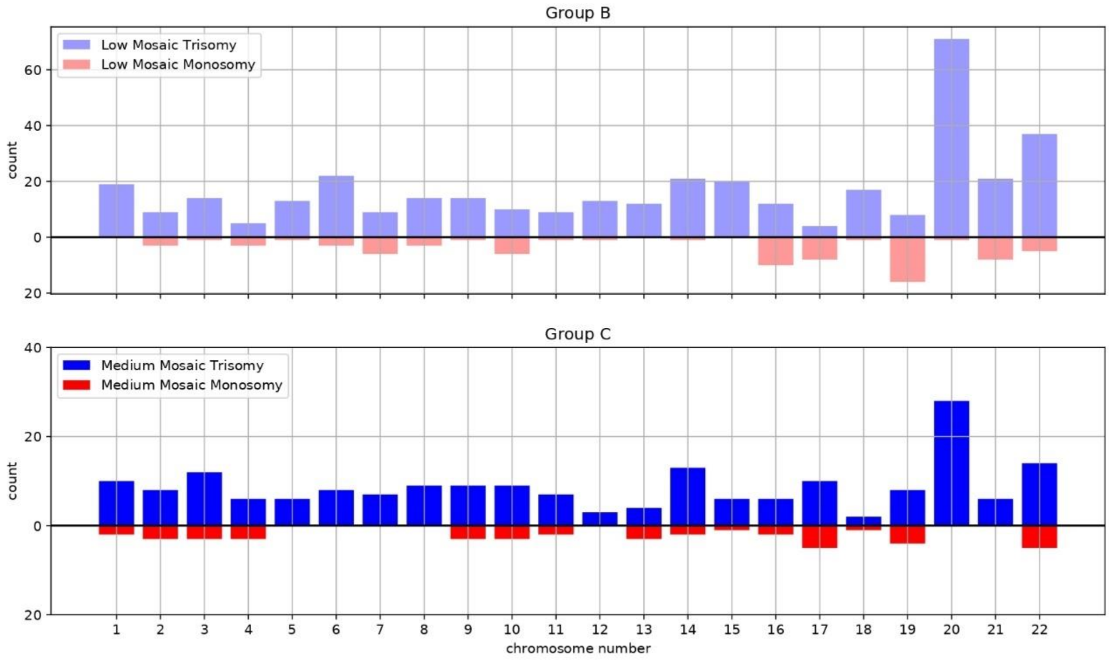

Figure S2 – Euploidy with biparental inheritance in children born from 'mosaic' embryo transfer – full genetic characterization. A) Cumulative AB heterozygous SNPs in the child, where the parents were of genotypes AA and BB (AABBAB- upper row) or BB and AA (BBAAAB – lower row) for all chromosomes. **B)** LogR and B allele frequencies for all chromosomes from a child born from Group C. The green line is the smoothed logR curve. For both panels, chromosome 6 is shown in **Figure 3**.

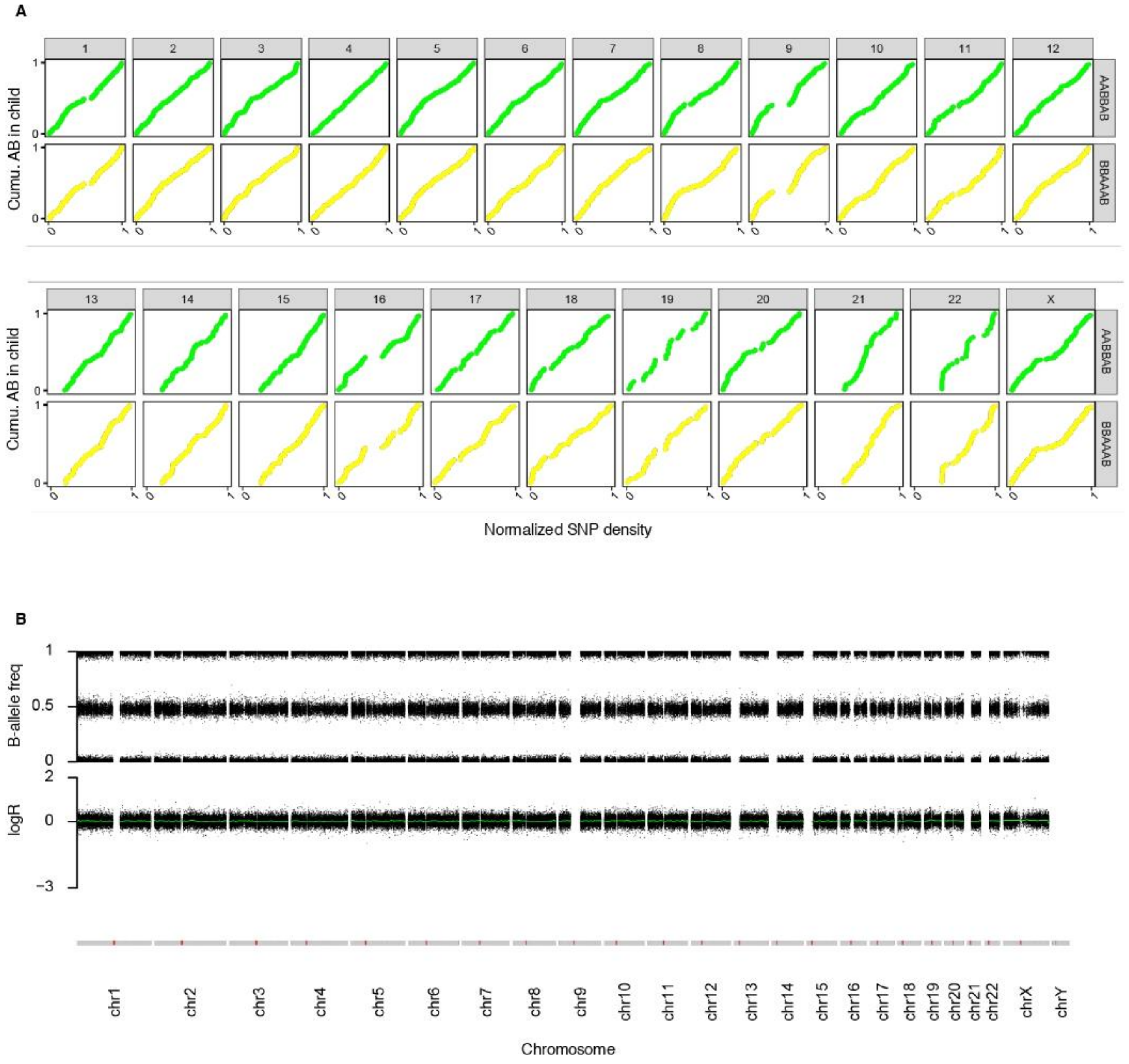

Figure S3 – Estimated impact on cumulative live birth rates in case putative mosaicism embryos are excluded from clinical use. Expected cumulative Live Birth Rate (LBR) per cycle with utilization of all embryos (euploid + putative mosaics) obtained per each cycle is shown in *green* line across the board of female age. *Brown* line projects the situation where putative moderate mosaic embryos are not utilized (- 7% for the observed model and -11% overall relative reduction for the projected model). *Orange* line depicts a scenario where all putative mosaics above 20% variability were excluded from transfer (-24% for the observed model and -36% overall relative reduction for the projected model). **A)** Observed relative reduction in cumulative LBR per cycle based on actual data from this trial. **B)** Optimistic model accounting for the combined probability of LBR in the case all transferable embryos are utilized. This modelling is based on the optimistic scenario, assuming that couples with embryos available for transfer who had not already returned for a subsequent replacement cycles would have the same chance of a pregnancy resulting in a live birth as the recorded LBR per embryo transfer in the whole euploid category (i.e., 43%). The mosaicism incidence is plotted based on the rate observed in the trial.

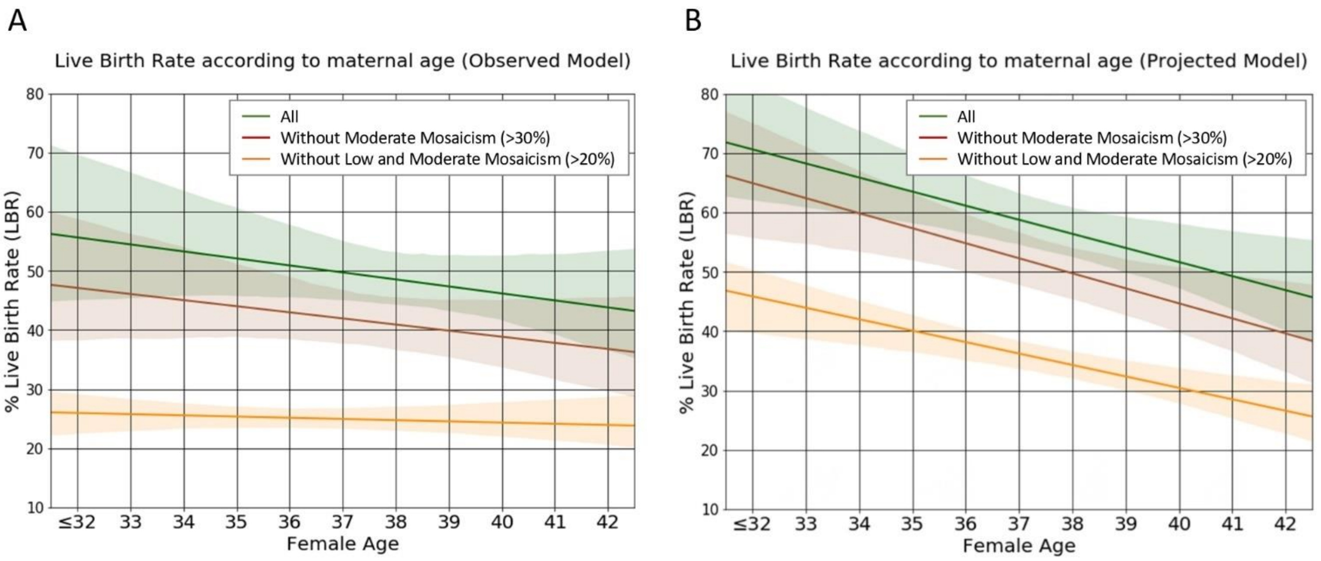

SUPPLEMENTAL TABLES

Table S1 – Demographic data of couples enrolled in the trial.

**DEMOGRAPHIC DATA OF ENROLLED COUPLES**	
Participants, n	783
Embryo transfer procedures, n	897
Mean female age (SD)	37.50 (± 3.3)
BMI female	21.7 (± 2.7)
FSH (mIU/mL), mean (± SD)	8.0 (± 4.2)
AMH (ng/mL), mean (± SD)	2.8 (± 2.9)
**Indication to PGT-A per cycle**	
Advanced Maternal Age (AMA), n (%)	576/783 (73.6%)
Repeated Implantation Failures (RIF), n (%)	32/783 (4.1%)
Repeated Pregnancy Loss (RPL), n (%)	28/783 (3.6%)
AMA + RIF, n (%)	27/783 (3.4%)
AMA + RPL, n (%)	13/783 (1.7%)
No Indication, n (%)	107/783 (13.7%)
**Protocol per cycle**	
Antagonist, n (%)	706/783 (90.2%)
Antagonist, n (%)	16/783 (2.0%)
DuoStim, n (%)	61/783 (7.8%)
**Semen**	
Ejaculated, n (%)	768/783 (98.1%)
Surgical, n (%)	14/783 (1.8%)
Donated, n (%)	1/783 (0.1%)
Sperm concentration [millions/ml], mean (± SD)	32.8 (± 26.1)
Sperm progressive motility [A+B%], mean (± SD)	38.7 (± 17.1)
Sperm morphology [% sperm with normal morphology], mean (± SD)	4.4 (± 2.6)
**Cycle data**	
Retrieved oocyte, mean (± SD)	9.1 (± 5.0)
2pn zygotes, mean (± SD)	6.7 (± 3.6)
Biopsied embryo [n], (mean ± SD)	2,874 (3.4 ± 1.9)
Euploid embryos, n (%)	1,774/2,874 (61.7%)
EUPLOID (<20%), n (%)	941 /2,874 (32.7%)
EUPLOID (20%-30%), n (%)	541/2,874 (18.8%)
EUPLOID (30%-50%), n (%)	292/2,874 (10.2%)
Aneuploid embryos (>50%), n (%)	1,100/2,874 (38.3%)

Table S2 – Additional mosaicism metrics and their association with single embryo transfer outcomes.

	Biochem Pregnancy Loss		Miscarriage		Live birth	
	YES	NO	YES	NO	YES	NO
Mean mosaicism rate<br>% (95%CI)	20.4%<br>(19.6-21.3)	22.6%<br>(20.3-24.9)	20.4%<br>(19.5-21.2)	20.8%<br>(18.1-23.5)	20.9%<br>(20.1-21.7)	20.4%<br>(19.6-21.3)
Mean number of mosaic chromosomes<br>(95%CI)	0.98<br>(0.59-1.37)	0.98<br>(0.83-1.14)	1.1<br>(0.54-1.68)	0.97<br>(0.80-1.13)	0.97<br>(0.81-1.13)	1.0<br>(0.86-1.15)
Incidence of complex mosaic<br>(>3 chr)<br>% (n) P-value (Y Vs N)	12.1%<br>(7/58)	13.4%<br>(59/440)<br>P=0.5	11.5%<br>(6/52)	13.7%<br>(53/388)<br>P=0.82	13.7%<br>53/386	12.5%<br>64/511<br>P=0.6
Incidence of complex mosaic<br>(>5 chr)<br>% (n) P-value (Y Vs N)	3.4%<br>(2/58)	5.2%<br>(23/440)<br>P=0.5	7.7% (4/52)	4.9%<br>(19/388)<br>P=0.33	4.9%<br>(19/386)	5.5%<br>(28/511)<br>P=0.8

Table S3 – Chromosome-specific analysis on the primary outcome measure. Projected and observed cumulative birth rates in cases where i) all embryos with NGS profiles with chromosomal copy number variations below 50% are transferred, ii) if embryos with >30% variation are not transferred and iii) if embryos with >20% variation are not transferred.

Chromosome involved in mosaicism		LBR .00	LBR 1.00	Total	Chromosome involved in mosaicism		LBR .00	LBR 1.00	Total
1	Count	21	17	38	12	Count	11	10	21
	% within chr_mosa_MAX	55.3%	44.7%	100.0%		% within chr_mosa_MAX	52.4%	47.6%	100.0%
	% within LBR	4.1%	4.4%	4.2%		% within LBR	2.2%	2.6%	2.3%
2	Count	16	12	28	13	Count	16	10	26
	% within chr_mosa_MAX	57.1%	42.9%	100.0%		% within chr_mosa_MAX	61.5%	38.5%	100.0%
	% within LBR	3.1%	3.1%	3.1%		% within LBR	3.1%	2.6%	2.9%
3	Count	18	14	32	14	Count	30	22	52
	% within chr_mosa_MAX	56.3%	43.8%	100.0%		% within chr_mosa_MAX	57.7%	42.3%	100.0%
	% within LBR	3.5%	3.6%	3.6%		% within LBR	5.9%	5.7%	5.8%
4	Count	10	4	14	15	Count	18	13	31
	% within chr_mosa_MAX	71.4%	28.6%	100.0%		% within chr_mosa_MAX	58.1%	41.9%	100.0%
	% within LBR	2.0%	1.0%	1.6%		% within LBR	3.5%	3.4%	3.5%
5	Count	11	11	22	16	Count	21	17	38
	% within chr_mosa_MAX	50.0%	50.0%	100.0%		% within chr_mosa_MAX	55.3%	44.7%	100.0%
	% within LBR	2.2%	2.8%	2.5%		% within LBR	4.1%	4.4%	4.2%
6	Count	27	25	52	17	Count	13	13	26
	% within chr_mosa_MAX	51.9%	48.1%	100.0%		% within chr_mosa_MAX	50.0%	50.0%	100.0%
	% within LBR	5.3%	6.5%	5.8%		% within LBR	2.5%	3.4%	2.9%
7	Count	10	6	16	18	Count	20	11	31
	% within chr_mosa_MAX	62.5%	37.5%	100.0%		% within chr_mosa_MAX	64.5%	35.5%	100.0%
	% within LBR	2.0%	1.6%	1.8%		% within LBR	3.9%	2.8%	3.5%
8	Count	20	12	32	19	Count	41	24	65
	% within chr_mosa_MAX	62.5%	37.5%	100.0%		% within chr_mosa_MAX	63.1%	36.9%	100.0%
	% within LBR	3.9%	3.1%	3.6%		% within LBR	8.0%	6.2%	7.2%
9	Count	17	16	33	20	Count	72	71	143
	% within chr_mosa_MAX	51.5%	48.5%	100.0%		% within chr_mosa_MAX	50.3%	49.7%	100.0%
	% within LBR	3.3%	4.1%	3.7%		% within LBR	14.1%	18.4%	15.9%
10	Count	18	15	33	21	Count	36	25	61
	% within chr_mosa_MAX	54.5%	45.5%	100.0%		% within chr_mosa_MAX	59.0%	41.0%	100.0%
	% within LBR	3.5%	3.9%	3.7%		% within LBR	7.0%	6.5%	6.8%
11	Count	8	4	12	22	Count	57	34	91
	% within chr_mosa_MAX	66.7%	33.3%	100.0%		% within chr_mosa_MAX	62.6%	37.4%	100.0%
	% within LBR	1.6%	1.0%	1.3%		% within LBR	11.2%	8.8%	10.1%
					Total	Count	511	386	897
						% within chr_mosa_MAX	57.0%	43.0%	100.0%
						% within LBR	100.0%	100.0%	100.0%

Table S4: Cumulative live birth rate per cycle data points according to the observed and projected model.

Female Age	Live Birth Rate according to maternal age (Observed Model)						Live Birth Rate according to maternal age (Projected Model)					
	All		Without moderate mosaicism (>30%)		Without low+ mod mosaicism (>20%)		All		Without moderate mosaicism (>30%)		Without low+mod mosaicism (>20%)	
	% (n)	95% CI	% (n)	95% CI	% (n)	95% CI	% (n)	95% CI	% (n)	95% CI	% (n)	95% CI
≤ 32	43.5 (27/62)	31.2-55.9	40.3 (25/62)	28.1-52.5	25.8 (16/62)	14.9-36.7	63.3 (39.3/62)	51.3-75.3	60.9 (37.8/62)	48.8-73.1	46.5 (28.8/62)	34.1-58.9
33	68 (17/25)	49.7-86.3	52 (13/25)	32.4-71.6	28 (7/25)	10.4-45.6	77.4 (19.3/25)	61.0-93.8	69.8 (17.4/25)	51.8-87.8	48.1 (12/25)	28.5-67.7
34	43.8 (21/48)	29.7-57.8	33.3 (16/48)	20.0-46.7	20.8 (10/48)	9.3-32.3	54.8 (26.3/48)	40.8-68.9	45.3 (21.8/48)	31.2-59.4	33.4 (16.1/48)	20.1-46.8
35	60 (39/65)	48.1-71.9	53.8 (35/65)	41.7-66.0	26.2 (17/65)	15.5-36.8	69.9 (45.4/65)	58.7-81.0	63.1 (41/65)	51.4-74.8	40.1 (26.1/65)	28.2-52.0
36	47.6 (59/124)	38.8-56.4	41.9 (52/124)	33.3-50.6	27.4 (34/124)	19.6-35.3	59.8 (74.2/124)	51.2-68.5	55.2 (68.4/124)	46.4-63.9	39.6 (49.2/124)	31.0-48.2
37	57.8 (48/83)	47.2-68.5	49.4 (41/83)	38.6-60.2	25.3 (21/83)	15.9-34.7	67.2 (55.8/83)	57.1-77.3	60.3 (50.1/83)	49.8-70.9	39.9 (33.1/83)	29.3-50.4
38	45.9 (50/109)	36.5-55.2	40.4 (44/109)	31.2-49.6	23.9 (26/109)	15.9-31.9	53.9 (58.7/109)	44.5-63.2	48.4 (52.7/109)	39.0-57.8	32.4 (35.3/109)	23.6-41.2
39	51.6 (49/95)	41.5-61.6	43.2 (41/95)	33.2-53.1	25.3 (24/95)	16.5-34.0	59.3 (56.4/95)	49.4-69.2	51.8 (49.2/95)	41.8-61.9	34.7 (32.9/95)	25.1-44.2
40	42.4 (36/85)	31.8-52.9	36.5 (31/85)	26.2-46.7	21.2 (18/85)	12.5-29.9	46.7 (39.7/85)	36.1-57.3	41.9 (35.6/85)	31.4-52.4	27.3 (23.2/85)	17.8-36.8
41	51.4 (36/70)	39.7-63.1	44.3 (31/70)	32.6-55.9	30 (21/70)	19.3-40.7	55.2 (38.6/70)	43.5-66.8	48.3 (33.8/70)	36.6-60.0	33.4 (23.4/70)	22.4-44.5
42	35.3 (12/34)	19.2-51.4	26.5 (9/34)	11.6-41.3	20.6 (7/34)	7.0-34.2	39.1 (13.3/34)	22.7-55.5	30.3 (10.3/34)	14.8-45.7	23.1 (7.9/34)	8.9-37.3

SUPPLEMENTAL METHODS: CLINICAL TRIAL REPORTING

Participant's inclusion/exclusion criteria and clinical trial flow-chart

Couples were included in the trial if below the age of 45 (female partner), using their own oocytes, undergoing ICSI for all oocytes and had at least one transferrable embryo available (euploid or low/medium-grade mosaic). Women with a history of unilateral oophorectomy, recurrent spontaneous abortion, diagnosis of the polycystic ovary syndrome, or uterine abnormality (e.g., Müllerian duct anomaly, adenomyosis, submucous myoma, intra uterine adhesion, or scarred uterus) were excluded. Women were also excluded if they had a chronic medical condition that has been associated with adverse pregnancy outcomes (i.e., endometriosis, autoimmune disorders such as severe systemic lupus erythematosus). All the couples were screened with the use of standard karyotyping, and those with numerical or structural uniform or mosaic abnormalities were excluded from the trial. Individuals showing unstable illnesses or medical conditions that may put their safety at risk were also excluded from the study (i.e., cancer, severe obesity). Cases where the only embryo available was of the worst morphological grade (according to the Gardner's criteria) were also excluded from the study. This criterion was introduced to mitigate an intrinsic bias, as euploid blastocysts of very poor morphological grade were shown to result in lower live birth and higher miscarriage rate compared to embryos with better morphology[1].

A total of 1,603 IVF cycles from 1,190 couples were assessed for eligibility from September 2018 through December 2019. Of these cycles, 266 were excluded from the study as 41 didn't produce any normally fertilised zygotes (41/1,603; 2.6%) and 225 produced blastocysts ineligible for biopsy (225/1,603; 14%). In addition, of the 1,337 cases undergoing trophectoderm biopsy and PGT-A, 490 were excluded as they led to all aneuploid embryos (490/1,337; 36.6%). Finally, a total of 783 couples were enrolled in this trial, 41 of which underwent two stimulation cycles. Overall, 824 stimulations led to 897 single-embryo transfers, with 50 couples receiving two SET and 7 couples receiving three SET. All remaining 676 couples received one SET at the time the enrolment to this trial was closed. Embryo morphology-based embryo selection led to the transfer of 484 uniform euploid embryos (Group A), 282 putative low mosaic embryos (Group B) and 131 putative moderate mosaic embryos (Group C) (**Figure 2**). Baseline characteristics and main IVF cycle outcomes of couples that entered the study are shown in **Table S1**.

Primary and secondary outcomes could be monitored for all cases, apart from mean gestational age at birth and mean birth weight which were obtained in 97% of cases. A minority of miscarriages could be characterized cytogenetically by product of conception (POC) analysis (n=4/52; 7.7%) and only 26 pregnancies underwent prenatal diagnosis

(PND;n=26/388, 6.7%). A total of 50 samples were collected from either putative mosaic (n=36) and uniformly euploid (n= 14) embryo-derived newborns. Of these, 38 passed QC and were selected for molecular testing follow-up involving postnatal karyotyping and genotyping. All remaining cases from putative mosaic embryos declined to participate in this phase of the study.

Description of primary and secondary outcome measures

The primary outcome measure was sustained implantation rate[3], defined as live birth rate (LBR) per transferred embryo according to the WHO and ICMART International Glossary on Infertility and Fertility Care[2]. The LBR is calculated as the number of newborns delivered on or after 22 weeks of gestation over the number of embryos replaced. In the event of a single-embryo transfer as occurred in this study for all cases, the metric is identical to delivery rate per transfer. The secondary outcome was miscarriage rate defined as the spontaneous loss of an intra-uterine pregnancy prior to 20 completed weeks of gestational age. This included the evaluation of pregnancy rate (PR), biochemical pregnancy (BP), clinical miscarriage (CM). Mean gestational age at birth and birth weight were also collected as additional neonatal outcomes. Adverse outcomes included the detection of chromosomal abnormalities, including uniparental disomy, in the miscarried product of conception (POC), during prenatal diagnosis (PND; amniocentesis/chorionic villi sampling, CVS) and/or at birth.

The implication of excluding putative mosaic embryos from clinical use has been evaluated in consideration of the potential loss of live births in a given IVF treatment cycle (Cumulative LBR, CLBR per cycle) assuming two scenarios: i) using actual data from embryo transfer in the study period excluding live births achieved from low and moderate mosaic embryos; ii) by modelling the optimistic scenario where all transferable embryos are replaced[4,5].

Outcome measures were described according to the International Glossary on Infertility and Fertility Care[2] and standard definitions:

Pregnancy rate (PR): The number of couples with positive serum level of β-human chorionic gonadotropin (βhCG) ($\geq$ 25 mIU/mL) per embryo transfer.

Live birth rate (LBR): The number of deliveries that resulted in at least one live birth per embryo transfer. Live birth is defined as the complete expulsion or extraction from a woman of a product of conception after 22 weeks of gestation, which, after such separation, breathes or shows any other evidence of life, such as heartbeat, umbilical cord pulsation or definite movement of voluntary muscles, irrespective of whether the umbilical cord has been cut or the

placenta is attached. In our study all embryo transfer procedures were of Single Embryo Transfer. Accordingly, LBR can be approximated as the number of live births divided by the number of SET procedures.

Implantation rate (IR): The number of gestational sacs observed by vaginal ultrasound at the 5th gestational week divided by the number of embryos transferred.

Clinical miscarriage rate (CMR)*:* Number of spontaneous pregnancy losses before week 20 in which a gestational sac/s was previously observed, per number of pregnancies, excluding ectopic pregnancy.

Biochemical pregnancy rate (BPR): Number of pregnancies diagnosed only by βhCG detection without a gestational sac visualized by vaginal ultrasound at the 5th week of pregnancy, per number of pregnancies.

Ectopic pregnancy rate (EPR): Number of pregnancies outside the uterine cavity, diagnosed by ultrasound, surgical visualization or histopathology, per number of pregnancies. For cumulative outcomes, we considered the clinical results obtained from all the embryo transfers performed in the same arm of the study up to 12 months follow-up.

Cumulative live birth rate (CLBR)*:* The number of deliveries that resulted in at least one live birth (as previously defined), per total number of couples receiving embryo transfer following the same type of transfer arm into which the couple was randomized for up to 12 months follow-up period. All clinical outcomes are presented as percentage (%).

Cumulative live birth rate per cycle (CLBR per cycle): the number of deliveries that resulted in at least one live birth (as previously defined) in a single ovarian stimulation cycle.

IVF treatment and embryology procedures

Standard IVF procedures were carried out for enrolled couples without any specific intervention apart from those specified. Ovarian stimulation and embryo culture and transfer were performed according to standard practice at each clinic. In all centres, embryo biopsy procedures were performed according to a previously published protocol[6]. Briefly, expanded blastocysts with or without herniating cells were submitted to TE biopsy. The embryo was anchored to the holding pipette maintaining the ICM at 7 o'clock orientation, allowing the biopsy to be performed at 2 o'clock and minimise interference with the ICM. A diode laser was employed to facilitate the opening of the zona pellucida and allow the passage of the biopsy pipette. Media was gently blown on the TE layer to detach the cells from the zona pellucida. Once detached, 3-10 cells were gently aspirated into the pipette and laser pulses were

directed to the junctions connecting the cells whilst moderate aspiration was applied. Once fully detached, the biopsied cells were released in the same drop, next to the embryo.

Tubing of biopsied cells was performed by briefly washing the specimen in PBS-based solution and transferring it into a 0.2mL sterile PCR tube with a final volume of <2μL.

The tubes were maintained at 4˚C (at the laboratory and during shipment) until arrival at the Igenomix Italy laboratory where they were processed and analysed. Biopsied embryos were cryopreserved using standard vitrification protocol employed by the specific clinic[7]. Euploid embryos were subsequently warmed using the standard warming protocol employed by the specific clinic and transferred to couples either in natural or stimulated embryo transfer cycles arranged as per routine in the specific clinic.

NGS analysis of TE biopsy samples

PGT-A analysis was performed using a semiautomated Next-generation sequencing (NGS) protocol (Ion Reproseq PGS Kit, Thermo Fisher Scientific, MA, USA) with Ion Chef™ equipment for library preparation and multiplexing up to 24-96 samples on a S5 XL sequencer (520 and 530 chips respectively). For individual samples, the most important QC parameters were i) the number of reads (required to be > 70,000 for PGT-A and > 120,000 for PGT-SR), ii) the dispersion/noise of the profile as measured by the mean absolute percent deviation (MAPD) (required to be < 0.3), and iii) the number of duplicates (required to be < 30%). A sample was considered informative if all these parameters were met.

Sequencing data obtained by the S5 sequencer were processed and transferred to Ion Reporter software for analysis (Thermo Fisher Scientific). Chromosome copy number values were calculated per each sample/chromosome and classified in a binary way: euploid (< 50% aneuploid) and aneuploid (≥ 50% aneuploid). Following the completion of all embryo transfer procedures included in the interim analysis, the Euploid category was further elaborated and the NGS data results subdivided in uniformly euploid (<20% aneuploid), low-putative mosaic (20-30%) and moderate putative mosaic (30-50%). This elaboration was independent from any proprietary diagnostic algorithm used in PGT-A laboratories. It primarily consists in the analysis of raw sequencing data with the specific protocol employed here without any chromosome-specific consideration. The NGS protocol used in this study has been extensively validated in our laboratories and shown to be capable of detecting mosaicism in fibroblast cell line

mixture models and diluted genomic DNA samples mimicking mosaicism[8]. Embryo transfer outcomes were then compared across the three different Euploid categories.

Reproducibility of PGT-A procedures and clinical outcomes consistency across the 5 participating centres was previously established[9]. The accuracy of the NGS protocol employed in this study was previously validated for mosaicism detection from cell line mixture models and embryo re-biopsies[8,10].

Additional data analysis and results from the clinical trials

We also evaluated if embryos showing putative mosaic profiles in multiple chromosomes were associated with different transfer outcomes. For this purpose, we assessed all possible metrics associated with chromosome copy number variations and main transfer outcomes (**Table S2**). Mean mosaicism rate (defined as average chromosome copy number value among putative mosaic chromosomes) was not increased in BPL: 20.4% (95%CI=19.6-21.3) vs 22.6% (95%CI=20.3-24.9); embryos resulting in miscarriage vs ongoing pregnancies: 20.4% (95%CI 19.5-21.2) vs 20.8% (95%CI=18.1-23.5), respectively; and for embryos resulting in LB vs embryos failing to implant: 20.9% (95%CI 20.1-21.7) vs 20.4% (95%CI=19.6-21.3), respectively. Mean number of mosaic chromosomes (defined as the average number of distinct putative mosaic chromosomes) was also similar between LBR and control group (**Table S2**).

Also, the absolute number of chromosomal profiles consistent with mosaicism, more than 3 or more than 5 per embryo (commonly defined as complex mosaics) was not associated with BPL (OR=1.00; 95%CI=0.85-1.18), miscarriage (OR=1.05; 95%CI=0.89-1.23) or LBR outcome (OR=0.98; 95%CI=0.91-1.07). Furthermore, we did not detect any putative mosaic chromosome-specific profile associated with LBR outcome (**Table S2**).

Genotyping analysis follow-up strategy of families with babies born following putative mosaic embryo transfer

We contacted all families achieving a live birth following the transfer of a putative mosaic embryo (n= 176). Of these, 36 accepted to enrol in the follow up study, all other families declined their participation to further studies. QC analysis of the collections revealed that 27 (75.0%) met acceptance criteria for all specimens in the trio. As a reference, we selected a random cohort of families that achieved live birth following the transfer of a uniformly euploid embryo. Of the 13 sample trios received, 11 (84.6%) passed QC and could be employed as controls.

Sample collection procedure was performed autonomously by consenting couples using sterile buccal swabs ("FLOQSwabs", Copan diagnostics) provided by Igenomix laboratory.

Buccal cells were collected by firmly pressing and rotating the swab against the inside of the inner cheek for 1 minute, using an up and down motion. The swab was then put back into the original tube, which was labelled with the individual's full name and date of birth. Specimens were sent to Igenomix Italy laboratory at room temperature and, upon arrival, immediately processed for DNA extraction.

SNP Genotyping analysis of trios was conducted double-blind. We genotyped DNA extracted from buccal swabs from both parents and the live born using the Illumina CytoSNP v12 array that were processed on a NextSeq (Illumina Inc.), following the instructions and quality control by the manufacturer. We used GenCall score of 0.75 rather than 0.15 for clinical tests to determine chromosome content using the logR and B allele frequencies. Additionally, to determine parental haplotypes in the child, we selected homozygous SNPs of opposite genotypes in the parents (AA and BB or vice versa, termed supporting SNPs) and plotted the cumulative AB genotype of the child at each SNP. The false discovery rate of the expected AB genotype is less than 0.002% based on the AB genotypes across SNPs where the parental genotypes were the same (e.g., AA and AA for maternal and paternal genotypes, but AB in the child). The code for SNP array based UPD and mosaicism analysis of trios is publicly available at the following link: https://github.com/Meiomap/TrioAnalysis.

Estimated impact on cumulative live birth rates in case low and medium grade mosaicism embryos are excluded from clinical use.

The implication of excluding putative mosaic embryos from clinical use has been evaluated in consideration of the potential loss of live births in a given IVF treatment cycle (CLBR per cycle). The cumulative LBR for a complete cycle is defined as the chance of live birth from an ovarian stimulation cycle including all subsequent FETs from that cycle [3]. Two scenarios were analysed: i) using actual data from embryo transfer in the study period excluding live births achieved from low and moderate mosaic embryos; ii) by modelling the optimistic scenario where all transferable embryos are replaced. For this second approach, a probabilistic projection was computed accounting for all euploid embryos with or without putative mosaic embryos produced from a single ovarian stimulation cycle and considering the combined probability of achieving a live birth based on the available embryos. In this model, the CLBR per cycle was computed by an optimistic approach, that is assuming that all available embryos are transferred

in a given cycle and with a defined probability of success. Live birth rate per euploid or mosaic embryo was the actual value observed in the study across the three study groups (i.e., 43%; **Figure S2**). The observed and projected CLBR per cycle analysis (with and without the clinical use of putative mosaic embryos) is shown across all female ages.

SUPPLEMENATAL REFERENCES

1. Cimadomo, D. et al. Looking past the appearance: a comprehensive description of the clinical contribution of poor-quality blastocysts to increase live birth rates during cycles with aneuploidy testing. Human reproduction (Oxford, England) 34, 1206–1214 (2019).

2. Zegers-Hochschild, F. et al. The International Glossary on Infertility and Fertility Care, 2017. Human reproduction (Oxford, England) 32, 1786–1801 (2017).

3. Fischer, C. & Scott, R. T. Three simple metrics to define in vitro fertilization success rates. Fertility and sterility 114, 6–8 (2020).

4. Smith, A. D. A. C., Tilling, K., Nelson, S. M. & Lawlor, D. A. Live-Birth Rate Associated With Repeat In Vitro Fertilization Treatment Cycles. JAMA 314, 2654–2662 (2015).

5. Malizia, B. A., Hacker, M. R. & Penzias, A. S. Cumulative live-birth rates after in vitro fertilization. The New England journal of medicine 360, 236–43 (2009).

6. Capalbo, A. et al. Correlation between standard blastocyst morphology, euploidy and implantation: an observational study in two centers involving 956 screened blastocysts. Human reproduction (Oxford, England) 29, 1173–81 (2014).

7. Ubaldi, F. M. et al. Reduction of multiple pregnancies in the advanced maternal age population after implementation of an elective single embryo transfer policy coupled with enhanced embryo selection: pre- and post-intervention study. Human reproduction (Oxford, England) 30, 2097–106 (2015).

8. García-Pascual, C. M. et al. Optimized NGS Approach for Detection of Aneuploidies and Mosaicism in PGT-A and Imbalances in PGT-SR. Genes 11, (2020).

9. Capalbo, A. et al. Consistent and reproducible outcomes of blastocyst biopsy and aneuploidy screening across different biopsy practitioners: a multicentre study involving 2586 embryo biopsies. Human reproduction (Oxford, England) 31, 199–208 (2016).

10. Girardi, L. et al. Incidence, Origin, and Predictive Model for the Detection and Clinical Management of Segmental Aneuploidies in Human Embryos. The American Journal of Human Genetics 106, 525–534 (2020).
