# Supplementary material for: Mosaic human preimplantation embryos and their developmental potential in a prospective, non-selection clinical trial
Source: Am J Hum Genet. 2021 Nov 18;108(12):2238–47. doi: 10.1016/j.ajhg.2021.11.002 (PMC8715143; doi:10.1016/j.ajhg.2021.11.002)
Supplement: Document S2. Article plus supplemental information [file mmc2.pdf]

ARTICLE

Mosaic human preimplantation embryos and their developmental potential in a prospective, non-selection clinical trial

Antonio Capalbo,[1,*] Maurizio Poli,[1] Laura Rienzi,[2] Laura Girardi,[1] Cristina Patassini,[1] Marco Fabiani,[1] Danilo Cimadomo,[2] Francesca Benini,[3] Alessio Farcomeni,[4] Juliana Cuzzi,[5] Carmen Rubio,[6,7] Elena Albani,[8] Laura Sacchi,[8] Alberto Vaiarelli,[2] Matteo Figliuzzi,[1] Necati Findikli,[9,10] Onder Coban,[11] Fazilet K. Boynukalin,[12] Ivan Vogel,[13] Eva Hoffmann,[13] Claudia Livi,[3] Paolo E. Levi-Setti,[8] Filippo M. Ubaldi,[2] and Carlos Simón[6,7,14,15]

Summary

Chromosome imbalance (aneuploidy) is the major cause of pregnancy loss and congenital disorders in humans. Analyses of small biopsies from human embryos suggest that aneuploidy commonly originates during early divisions, resulting in mosaicism. However, the developmental potential of mosaic embryos remains unclear. We followed the distribution of aneuploid chromosomes across 73 unselected preimplantation embryos and 365 biopsies, sampled from four multifocal trophectoderm (TE) samples and the inner cell mass (ICM). When mosaicism impacted fewer than 50% of cells in one TE biopsy (low-medium mosaicism), only 1% of aneuploidies affected other portions of the embryo. A double-blinded prospective non-selection trial (NCT03673592) showed equivalent live-birth rates and miscarriage rates across 484 euploid, 282 low-grade mosaic, and 131 medium-grade mosaic embryos. No instances of mosaicism or uniparental disomy were detected in the ensuing pregnancies or newborns, and obstetrical and neonatal outcomes were similar between the study groups. Thus, low-medium mosaicism in the trophectoderm mostly arises after TE and ICM differentiation, and such embryos have equivalent developmental potential as fully euploid ones.

Introduction

Aneuploidy in human conceptions is the leading cause of embryo implantation failure, pregnancy loss, and congenital disorders in live-born infants. Although chromosome segregation errors in oocytes are its major cause,[1] recent observations suggest that aneuploidy can also arise significantly during preimplantation embryonic divisions.[2] Aneuploidies due to meiotic errors are present uniformly throughout the embryo, whereas those with a mitotic origin (i.e., post-fertilization) give rise to cell lineages with different chromosomal content (embryonic mosaicism). Understanding the incidence of mosaicism and the distribution of aneuploid cells throughout the embryo is important because genetic testing of preimplantation embryos and prenatal fetuses is carried out, respectively, on a trophectoderm biopsy or samples of placental tissue originated from it, rather than the cell lineage programmed to form the embryo proper (i.e., inner cell mass (ICM), ICM).

With the introduction of high-resolution next-generation sequencing (NGS) protocols, chromosomal mosaicism has been reported in up to 20% of clinical trophectoderm (TE) biopsies[3] (Figure 1A), which consist of a sample of 3 to 10 cells. Recent large genome-wide non-invasive prenatal screening (NIPS) studies performed at 12 weeks of gestation have shown that the presence of confined placental mosaicism (CPM) explains a significant fraction of false-positive cases of rare autosomal trisomies (RATs).[4,5] Yet, the prevalence of chromosomal mosaicism in human pregnancies is reported in fewer than 0.3% of prenatal tests (e.g., by amniocentesis or by NIPS).[6] On the basis of experiments in murine models, the sharp drop in mosaicism between pre- and post-implantation stages has been explained by the selective elimination of aneuploid cells through competitive growth of euploid cells or apoptosis of the abnormal cellular clones.[7,8] In humans, the prevalence and developmental potential of mosaic embryos consisting of both diploid and aneuploid cells remain the subject of intense debate.[9] This is largely due to a paucity of studies that have assessed mosaicism across the whole embryo; as a result, our understanding of the distribution of aneuploid cells is limited. One major clinical impact of this shortcoming has been that fewer than 3% of mosaic embryos are being used in in babies born after in vitro

[1]Igenomix, Reproductive Genetics, Marostica 36063, Italy; [2]Clinica Valle Giulia, GeneraLife IVF, Rome 00197, Italy; [3]DEMETRA, GeneraLife IVF, Florence 50141, Italy; [4]Department of Economics and Finance, University of Rome “Tor Vergata,” Rome 00133, Italy; [5]Igenomix US & Canada, Miami, FL 33126, USA; [6]Igenomix, Valencia 46980, Spain; [7]Igenomix Foundation, Reproductive Genetics, Valencia 46980, Spain; [8]Istituto di Ricovero e Cura a Carattere Scientifico, Humanitas Research Hospital, Division of Gynecology and Reproductive Medicine, Fertility Center, Rozzano (Milan) 20089, Italy; [9]Bahceci Fulya IVF Centre, Embryology Laboratory, Istanbul 34394, Turkey; [10]Department of Biomedical Engineering, Beykent University, Istanbul 34398, Turkey; [11]British Cyprus IVF Hospital, Nicosia 2681, Cyprus; [12]Bahceci Fulya IVF Centre, Infertility Clinic, Istanbul 34394, Turkey; [13]Danish National Research Foundation Center for Chromosome Stability, Department of Cellular and Molecular Medicine, Faculty of Health and Medical Sciences, University of Copenhagen, Copenhagen 2200, Denmark; [14]Valencia University and INCLIVA, Department of Obstetrics and Gynecology, Valencia, 46010, Spain; [15]School of Medicine, Department of Obstetrics and Gynecology, Harvard University, Cambridge, MA 02115, USA
*Correspondence: antonio.capalbo@igenomix.com
https://doi.org/10.1016/j.ajhg.2021.11.002.
© 2021 The Author(s). This is an open access article under the CC BY license (http://creativecommons.org/licenses/by/4.0/).

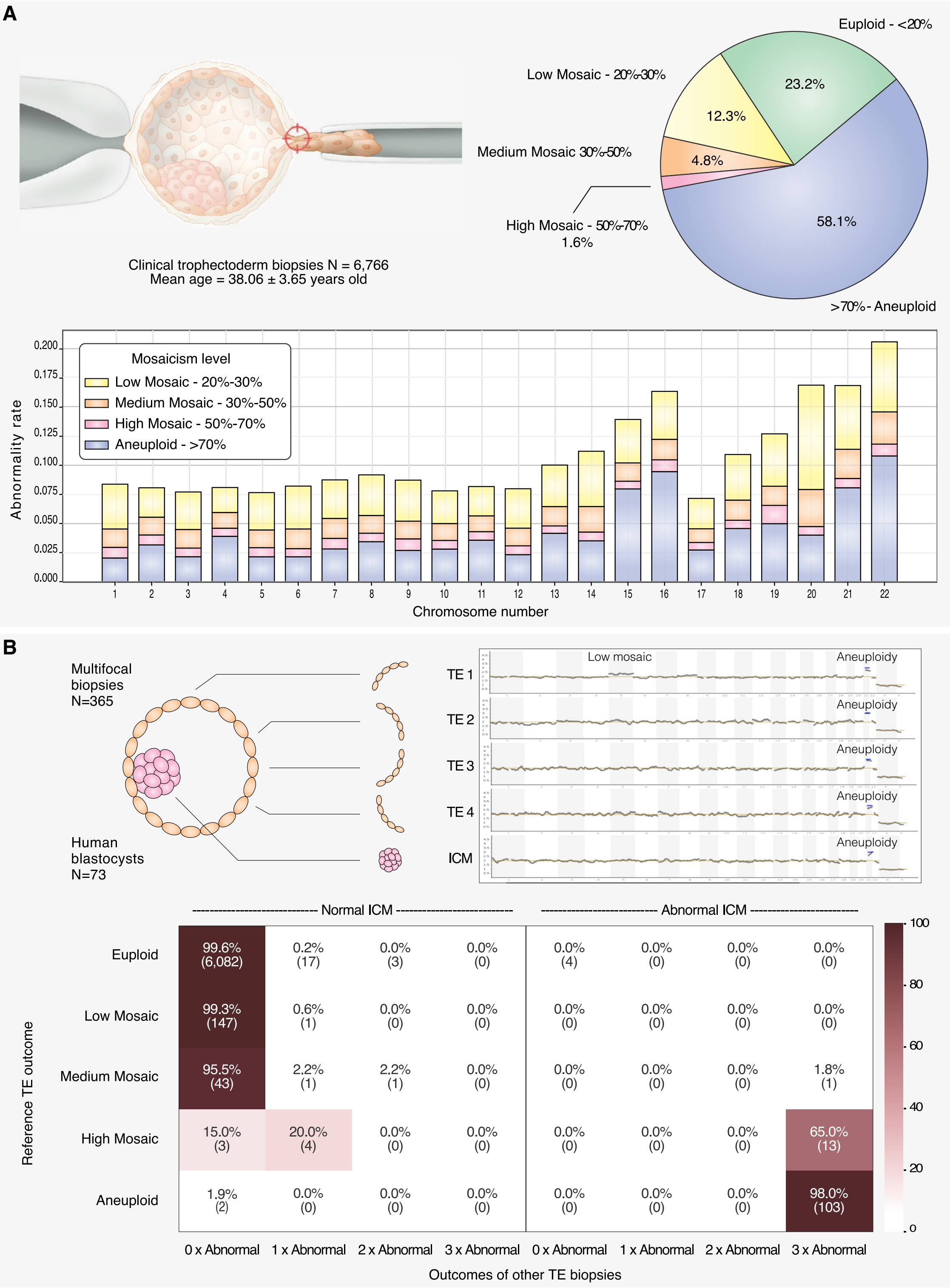

Figure 1. Aneuploidy incidence in clinical trophectoderm biopsies and embryonic concordance study
(A) The pie chart represents the distribution of aneuploidy categories observed in 6,766 clinical trophectoderm (TE) biopsies analyzed in our PGT-A diagnostic setting (classified according to the most severe abnormality across all chromosomes). The stacked bar chart represents the incidence of each aneuploidy category at the chromosome level.
(B) Top left: 73 human blastocysts were disaggregated into five portions: four TE biopsies and the inner cell mass (ICM) biopsy. Top right: examples of PGT-A plots displaying a low mosaic configuration confined to TE1 and a uniform aneuploidy detected in all portions. Bottom: this heatmap shows diagnostic concordance rates per chromosome on the basis of 73 embryos with five biopsies each (365 embryo biopsies), leading to 6,424 comparisons (73 embryos × 22 autosomal chromosomes × 4 permutations of reference biopsy). One of the four TE biopsies is considered as a reference, whereas the remaining biopsies (three TE + ICM) are used for verifying the outcome of the reference. Based on its copy-number result, each autosomal chromosome within the reference biopsy is classified into one of five

(legend continued on next page)

fertilization (IVF) treatment[10–12] as a result of concerns that the aneuploidy detected in the TE might affect the ICM and, therefore, potentially the baby. However, mosaicism is not generally detected at increased rates IVF treatment,[13–15] suggesting that aneuploid cells in mosaic preimplantation embryos do not contribute toward the genetic make-up of live-born infants. Consistent with this hypothesis, a small study of mosaic embryos showed they are capable of giving rise to healthy live births.[16]

Larger, retrospective studies have concluded that mosaic embryos have a lowered reproductive potential.[10,17] Such retrospective data, however, are affected by a strong selection bias. In particular, retrospective analyses do not take into account the fact that mosaic embryos are transferred as a last option and, consequently, that their reproductive performance is often measured on a highly selected subpopulation of women who had previous failed implantations with euploid embryos. Mosaic embryos are also transferred in those individuals producing only aneuploid embryos, introducing again a strong selection bias toward a poor-prognosis population. For example, in the largest retrospective study published to date where the reproductive competence of putative mosaic embryos was assessed, 94.6% of cases involving mosaic-embryo transfer were included because no uniformly euploid embryos were available.[17] Accordingly, the reproductive potential of mosaic embryos remains to be tested in a robust, well-powered prospective, non-selection clinical trial.

Here, we have first investigated the prevalence and distribution of aneuploid cells in the largest dataset of disaggregated human blastocysts currently available. Our findings show that in the majority of cases where mosaicism is detected in TE biopsies, this is due to a few aneuploid cells that originate from the trophectoderm tissue and give rise to a low- to medium-grade mosaicism configuration. To provide exhaustive evidence on the effects that highly localized aneuploid cells have on a mosaic embryo's clinical performance, we carried out a prospective non-selection study allowing an unbiased comparison between uniformly euploid and mosaic embryos in terms of reproductive potential and chromosomal normalcy between uniformly euploid and mosaic embryos, providing exhaustive evidence related to their clinical performance and the ensuing offspring. We report that putative mosaic embryos show clinical outcomes similar to those of uniformly euploid embryos and that there are not any significant implications for pregnancy and live-birth outcomes or the offspring's health. The clinical data generated from this trial will clearly resolve major concerns related to the management of mosaicism findings in preimplantation genetic testing for aneuploidies (PGT-A) and will be of fundamental importance for helping many infertile individuals to make more informed reproductive decisions.

Materials and methods

Assessment of mosaicism incidence and prevalence in human blastocysts

Data from clinical cases, consisting of 6,766 clinical TE biopsies performed in 2020 and 2021, were recovered so that the incidence of different aneuploidy categories observed in the diagnostic setting of PGT-A could be computed. A total of 91 blastocyst-stage human embryos were donated to research at the British Cyprus IVF Hospital under ethical-committee approval obtained from the institutional review board at Near East University (project number YDU/2018/64-685). Approved informed-consent forms were signed by all the individuals donating their embryos to this study. Embryos were warmed, and ICM biopsies were isolated by a previously validated mechanical method.[18] The TE was then disaggregated in four equally sized portions. Blinded NGS analysis was performed on all re-biopsies, and results were analyzed so that the prevalence and distribution of abnormal cells in the remaining embryonic sections could be assessed according to the original estimated mosaicism rate in the reference TE biopsy (see supplemental methods).

Clinical trial design and participants

We conducted a multicenter, double-blinded, non-selection trial (trial registration NCT03673592) involving consecutive IVF with blastocyst-stage PGT-A treatments followed by frozen euploid, low-grade, or medium-grade mosaic single-embryo transfer (SET). Women eligible for participation were below the age of 45, had autologous oocytes, were undergoing intra-cytoplasmic sperm injection (ICSI) for all oocytes, and had at least one transferable embryo available (euploid or low- to moderate-grade mosaic). Treatment cycles were excluded from the study if the embryo to be transferred showed the worst morphological grade according to an adaptation of Gardner's criteria[19] or if the female partner had a chronic medical condition associated with adverse pregnancy outcomes. The purpose this criterion was to mitigate an intrinsic bias; euploid blastocysts of very poor morphological grade were shown to result in lower live-birth and higher miscarriage rates than embryos with better morphology.[20] IVF procedures were carried out according to standard practices employed at each clinic (supplemental methods).

The trial was conducted in compliance with the International Conference on Harmonisation and the Declaration of Helsinki. The protocol was approved by the institutional review board of Clinica Valle Giulia, Rome (September 3, 2018) and the Humanitas

categories (euploid, low-grade mosaic [20–30%], medium-grade mosaic [30–50%], high-grade mosaic [50–70%], or aneuploid; rows), whereas the verification biopsies are classified in two categories (normal [<50%] or abnormal [>50%]). The heatmap shows concordance rates between the reference TE and the three verification TE biopsies (columns), given the outcome of the reference (rows). This analysis is split into two maps depending on whether the ICM is normal (left) or abnormal (right). For instance, the cell in the first column, second row, indicates that if a chromosome is detected at a low-grade mosaic configuration in the reference TE biopsy, there is a 99.3% probability that a normal diagnostic outcome is detected in all four other verification biopsies. The risk of chromosomal abnormalities in the four remaining embryonic portions is rare and similar when the reference biopsy shows a euploid, low-grade mosaic, or medium-grade mosaic outcome.

Research Hospital Ethics Committee, Rozzano (code 477/19). First participants were recruited beginning September 20, 2018. All the individuals eligible for the study provided written informed consent before starting ovarian stimulation. Inclusion in the study population was validated at the time of transfer, when all acceptance criteria were confirmed (i.e., availability of non-aneuploid embryos). The trial was registered with ClinicalTrials.gov as NCT03673592.

Intervention

After TE biopsy and NGS-based chromosomal analysis, a diagnostic report on embryos' chromosomal status was sent to the clinical sites (supplemental methods). Embryos showing a low or moderate degree of chromosomal mosaicism were blindly reported as euploid without distinction from uniformly euploid embryos. Among those reported as euploid, embryos were selected for SET on the basis of standard morphological features, providing a blinded allocation of the participants into the three main categories "euploid" (group A), "low-grade mosaic" (group B; 20–30% aneuploid cells), and "medium-grade mosaic" (group C; 30–50% aneuploid cells). Cases were followed up on during the post-transfer, gestational, and postnatal periods. The chromosomal status of 38 newborns derived from the transfer of putative mosaic embryos was investigated via single-nucleotide polymorphism arrays (SNPa genotyping) on saliva samples collected from the newborns and their parents. Genotyping data of the trios were used for investigating any potential instance of mosaicism or uniparental disomies (UPDs) in the offspring. Details of the genotyping protocol are reported in the supplemental methods.

Outcomes

The primary outcome measure was sustained implantation rate (the probability that any transferred embryo will implant and progress to delivery),[21] defined as live-birth rate (LBR) per transferred embryo according to the World Health Organization and International Committee for Monitoring Assisted Reproductive Technologies International Glossary on Infertility and Fertility Care.[22] The LBR was calculated as the number of newborns delivered on or after 22 weeks of gestation over the number of embryos replaced. In the event of a SET, as occurred in this study for all cases, the metric is identical to delivery rate per transfer. The secondary outcome was miscarriage rate, defined as the spontaneous loss of an intra-uterine pregnancy prior to 20 completed weeks of gestational age. This included the evaluation of pregnancy rate (PR) and biochemical pregnancy (BP). Mean gestational age at birth and birth weight were collected as neonatal outcomes. Adverse outcomes were determined by the detection of chromosomal abnormalities, including uniparental disomy, in the miscarried product of conception (POC) during prenatal diagnosis (PND; amniocentesis/chorionic villi sampling CVS) and/or at birth.

The implication of excluding putative mosaic embryos from clinical use has been evaluated in consideration of the potential loss of live births in a given IVF treatment cycle (cumulative LBR, CLBR per cycle) by two different methods: (1) using actual data from embryo transfer in the study period but excluding live births achieved from low- and moderate-mosaic embryos and (2) by modeling the optimistic scenario where all transferable embryos are replaced. A complete description of the outcome of these analyses is reported in the supplemental methods.

Statistical analysis

The primary endpoint for this analysis was the non-inferiority of LBR when euploid and mosaic embryos were compared. Assuming an LBR of 45% for uniformly euploid embryos versus 42.5% for low- or moderate-degree mosaics,[2] a 1:1 sampling ratio for the two groups, and a planning non-inferiority margin of 7.5%,[23,24] we calculated that 421 embryos per group would guarantee the power of at least 90% for a significance level set at 5%. The same sample size was sufficient for targeting non-inferiority claims in the miscarriage rate. Assuming a 10% miscarriage rate for uniformly euploid embryos and 15% for moderate to low mosaics, and setting a significance level at 5%, we also found the sample size had >90% power to assess non-inferiority in the miscarriage rate between control and test groups; the non-inferiority margin was 2%.

Data are expressed as mean ± standard deviation or percentages as appropriate. Proportions were compared via the chi-square test or Fisher exact test for 2 × 2 contingency tables. The non-inferiority endpoint was set as the 95% CI for difference in proportions lying below the planned margin. In addition to computing confidence intervals and p values for the difference in proportions, we computed the odds ratios (ORs) and adjusted odds ratios (AORs) of mosaicism for LBR, PR, IR, MC, and BP through logistic regression models. In multivariate analyses, ORs were adjusted for female age, male age, center, morphology of the blastocyst, day of the biopsy, number of previous implantation failures, previous miscarriages, previous live birth, infertility indication, and sperm origin (ejaculated versus surgical). All other tests were two tailed. Data had no missing values for variables involved in the primary analysis. A complete case approach was used for secondary analyses. All analyses were conducted with SPSS v. 21 and R v. 3.5.1.

Results

Incidence and prevalence of chromosomal mosaicism on blastocyst-stage human embryos

To shed light on the incidence and prevalence of chromosomal mosaicism in human preimplantation embryos, we first analyzed a large historical dataset of 6,766 embryos, where a clinical trophectoderm (cTE) biopsy had been processed with NGS technology. This approach could detect a mosaicism rate as low as 20% in cell line mixture models.[25] This clinical dataset of cTE biopsies (n = 6,766; mean maternal age n = 38.06; SD 3.65) revealed an incidence of diploid-aneuploid mosaicism of 18.7%; smaller chromosomes were more frequently represented than others (Figure 1A).

Next, we investigated the prevalence of mosaicism and its clonal distribution in 91 unselected human blastocysts that were donated for research. We divided the trophectoderm into four biopsies and isolated the ICM by using a validated mechanical method able to retrieve ICM specimens free of TE contamination (Figure 1B).[18] The collection of all five samples from the same blastocyst was successful for 75 out of 91 embryos. Blinded NGS analysis generated an informative result for all five samples in 73 embryos, allowing robust diagnosis for each of the four TE biopsies and the ICM. Chromosomal mosaicism rates were calculated for each of the five blastocyst biopsies,

and the concordance rate between the reference TE biopsy and the remaining four embryonic portions (including the ICM) was assessed on the basis of permutation analysis (Figure 1B). Using one TE biopsy as a reference, we found that chromosomal abnormalities in the ICM were extremely rare and not statistically different irrespective of whether the reference biopsy had been classified as euploid or low- to medium-grade mosaic (p = 0.14). In these cases, segregation patterns were confined to a single portion of the TE and rarely affected the ICM **(**Figure 1B). In contrast, high-grade mosaicism (50–70%) in a single TE sample was commonly associated with uniform aneuploidy throughout the embryo (including the ICM) in 65% of cases (13 out of 20 embryos; 95% CI = 43–82%; Figure 1B). We conclude that, in the vast majority of cases, the detection of low- to medium-grade mosaicism in one TE biopsy reflects a status of highly confined aneuploidy rather than its random distribution throughout the embryo.

Prospective non-selection clinical-trial results

Our data suggest that low- to medium-grade mosaic embryos might have developmental potential similar to that of euploid ones. So far, clinical outcomes of mosaic embryos have only been compared retrospectively in selected subpopulations of individuals who failed to get pregnant with previous transfers of euploid embryos or who only had one putative mosaic embryo available and were therefore of poor prognosis.[7,10–12] To produce unbiased evidence of the reproductive potential of low- to medium-grade mosaic embryos, we carried out a multicenter double-blinded non-selection clinical trial involving 1,190 couples and 1,603 IVF cycles (trial registration number NCT03673592). All cycles included blastocyst-stage preimplantation genetic testing for aneuploidy (a single clinical TE biopsy) and were initiated on September 20th, 2018 and ended on December 31st, 2019 (Figure 2). Embryos showing low- and medium-grade chromosomal mosaicism were blindly reported (non-selection) as euploid and transferred via SET based on standard morphological features.[19] Embryos were blindly allocated to three main categories: euploid (group A), low-grade mosaic (group B) (20–30% aneuploid cells), and medium-grade mosaic (group C) (30–50% aneuploid cells). The primary outcome measure was sustained implantation rate through delivery (presence of a viable pregnancy after 20 weeks of gestation), measured as the live birth rate (LBR), defined by the WHO as live births per embryos transferred. The secondary outcome measure was miscarriage rate, defined by loss of an intra-uterine pregnancy prior to the 20 completed weeks of gestational age. The distribution of mosaic chromosome types across each group of transferred mosaic embryos is reported in Figure S1**.** Baseline characteristics of study participants and main effects of treatments on those participants' IVF cycles were similar among the three groups and are shown in Table S1.

We found no evidence that low- or medium-grade mosaicism affected live birth rates among 484 uniform euploid embryos (group A), 282 low-degree mosaic embryos (group B), or 131 medium-degree mosaic embryo transfers (group C) included in the primary analysis, which was powered for the LBR as a primary outcome. LBRs of uniformly euploid embryos and low- and moderate-grade mosaic embryos were 43.4% (95% CI = 38.9–47.9%), 42.9% (95% CI = 37.1–48.9%), and 42% (95% CI = 33.4–50.9%), respectively (Table 1). The fact that the confidence interval for the difference fell below the planned 7.5% margin (95% CI = −5.7–7.3%) shows that the non-inferiority endpoint for the primary outcome measure was met, suggesting similar reproductive outcomes for euploid and low- and moderate-grade mosaic embryos. No difference was observed in miscarriage rates (OR = 0.89; 95% CI = 0.50–1.55; p = 0.69), providing additional support for chromosomal normalcy of pregnancies from low- and medium-grade mosaic embryos (Table 1). Additionally, the number of chromosomes showing a mosaic configuration (commonly referred as complex mosaic) was also not associated with any of the outcomes investigated (see Table S2). At a multivariate-analysis level, an effect on LBR was observed for poor-quality blastocyst morphology (adjusted odds ratio (AOR) = 0.56 compared to the top-quality category, 95% CI = 0.35-0.89; p = 0.0146), day of biopsy (e.g., day 5 versus day 6) (AOR = 0.68 per day, 95% CI = 0.51–0.90, p = 0.008), and surgical origin of sperm (AOR = 0.158, 95% CI = 0.04-0.75, p = 0.020).

Although our study was not powered to detect mosaicism in pregnancies, we nevertheless investigated potential mosaicism by conducting follow-up analysis of all the available products of conceptions (POCs) after spontaneous miscarriages and elective prenatal diagnosis procedures. Miscarriage rates were similar in the three groups (Table 1). Four of the 52 miscarriage cases were analyzed by standard cytogenetics, all of which were euploid. Twenty-six sustained pregnancies (26 out of 388, 6.7%) underwent prenatal diagnosis by amniocentesis (group A = 15; group B = 6; group C = 3) or by chorionic villi sampling (group A = 2). These numbers were small because the reduction in the use of invasive prenatal diagnosis is one of the aims of PGT-A. All prenatal diagnoses showed euploid karyotype except in one pregnancy from group A (uniformly euploid), where confined placental mosaicism for trisomy 22 (20% abnormal cells) was detected at chorionic villi sampling analysis. However, subsequent follow-up amniocentesis displayed a normal euploid cytogenetic result.

At birth, obstetric and neonatal outcomes were similar between mosaic and euploid embryos (Table 1). One developmental abnormality was observed among the moderate-grade mosaicism cases (group C), where a baby was born with a diagnosis of Beckwith-Wiedemann Syndrome caused by hypomethylation in the region KvDMR/IC2.[26] This condition is not a diagnostic target of PGT-A nor

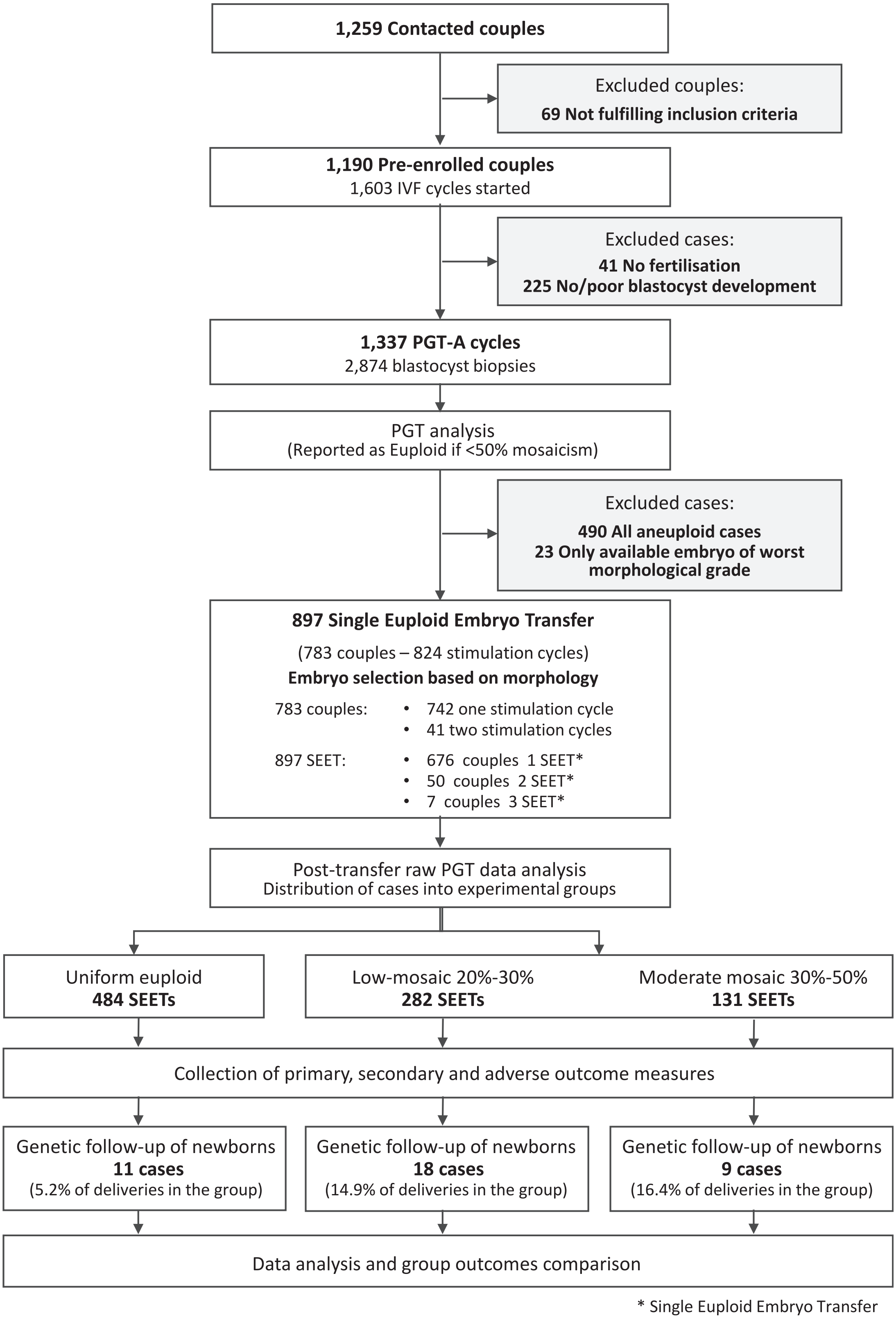

Figure 2. Study flow chart

due to chromosomal mosaicism. To further investigate any potential persistence of the abnormal cell line after mosaic embryo transfer, we carried out postnatal genotyping of newborns on a subset of 38 families willing to participate (9.8% of all newborns derived from the study; Figure 3). At genome-wide resolution, all genotyping tests showed

Table 1. Reproductive outcomes of euploid and mosaic embryos

	Group A: Euploid	Group B: Low-grade mosaic (20–30% variation)	Group C: Medium-grade mosaic (30–50% variation)	Adjusted OR (95% CI; p value)
Test sets, n	484	282	131	-
Positive pregnancy test, % (n)	55.8% (270/484)	55.0% (155/282)	55.7% (73/131)	0.98 (0.75–1.27; 0.86)
Biochemical pregnancy loss, % (n)	10.7% (29/270)	12.3% (19/155)	13.7% (10/73)	1.18 (0.69–2.02; 0.53)
Miscarriage, % (n)	12.0% (29/241)	11.0% (15/136)	12.7% (8/63)	0.89 (0.50–1.55; 0.69)
Live birth, % (n)	43.4% (210/484)	42.9% (121/282)	42.0% (55/131)	0.97 (0.74–1.26; 0.82)
Monochorial twins delivery, n	1	1	1	-
Gestational age, mean (95% CI)	38.4 (38.0–38.7)	38.2 (37.9–38.6)	38.1 (38.0–38.5)	-
Birth weight, mean (95% CI)	3,286 (3,200–3,371)	3,174 (3,080–3,267)	3,130 (2,950–3,310)	-

Biochemical pregnancy is defined by a positive pregnancy test. Implantation rate is defined as the number of gestational sacs observed by vaginal ultrasound at the fifth gestational week divided by the number of embryos transferred. Multiple pregnancy is defined by any scan with more than one heartbeat or gestational sac at the stage of clinical pregnancy (approximately 6 weeks). Miscarriage is defined as the loss of a clinical pregnancy, excluding ectopic pregnancies, before 20 weeks of gestation. A live birth is defined as a delivery that resulted in at least one live birth after 22 weeks of gestation. CI denotes confidence interval. "-" indicates not applicable.

fully normal karyotypes and the absence of uniparental ditype configuration in the cohort of babies born from mosaic-embryo transfer (Figure 3 and Figure S2).

To determine the clinical impact of not transferring low- and medium-grade mosaic embryos, we developed a theoretical model of cumulative treatment outcomes on the basis of the incidence of mosaicism and clinical outcomes from this trial (~43% LBR). If we model a scenario that considers the embryos transferred in the trial, an overall reduction of 24 and 7% in LBR would be expected if low- and medium-grade mosaic embryos or if medium-grade embryos only were removed, respectively (Figure S3A). If we consider an optimistic scenario that assumes all potentially available embryos are transferred for a given IVF treatment cycle, not transferring group B and C or group C alone would have resulted in an overall reduction in cumulative LBR of 36 and 11%, respectively (Figure S3B; source data from Tables S3 and S4).

Discussion

Since the introduction of NGS in preimplantation genetic testing for aneuploidy (PGT-A), there has been substantial debate over the incidence and the distribution of aneuploid cells throughout human preimplantation embryos and how this might affect developmental potential. In this study, we combined the assessment of both the distribution of aneuploid cells throughout human blastocysts and their impact on the embryo's reproductive potential. The latter parameter was investigated in a prospective multicenter, double-blind, non-selection trial aimed at assessing the clinical performance and safety of low- and medium-degree mosaic embryos by minimizing biases deriving from skewed populations' prognoses and embryo-prioritization strategies.

First, we assessed the distribution of aneuploid cells throughout blastocyst embryos by analyzing aneuploidy in 365 biopsies derived from 73 unselected blastocyst embryos. Our data support a model in which aneuploidy is confined to a small cluster of cells in the peripheral placental lineages (i.e., TE) at the peri- and early post-implantation stages. This results in low- and medium-grade mosaic embryos when a single cTE biopsy is taken and the signal from aneuploid cells is "averaged" over 3–10 cells. In effect, our disaggregation experiments suggest that once the aneuploid cells have been removed, the remaining embryo consists of euploid cells.

The subsequent prospective, non-selection clinical trial found no evidence of inferior performance of low- and medium-grade mosaic embryos with regards to pregnancy outcomes, including LBR, pregnancy loss, or chromosomal abnormalities in the pregnancy and in children (Table 1, Figure 3). In fact,[16] on the basis of the transfer of thousands of mosaic embryos carried out to date, the positive predictive value of a mosaic finding in blastocyst embryos by PGT-A has been confirmed in only a single case.[17,27] Accordingly, the evidence from our trial does not support the cascade of procedures commonly triggered by the reporting of mosaicism in PGT-A testing cases, including additional genetic counseling sessions, intensified anxiety and distress in treated individuals, higher costs, increased adoption of invasive prenatal diagnosis,[28,29] and its associated risk of iatrogenic abortion (0.3%).

Whereas low- and medium-grade mosaicism do not appear to affect live birth, the outcome of high-grade mosaic embryos is clearly a different matter given that 65% of high-grade mosaic embryos were extensively affected, including in the inner cell mass (Figure 1B). In this study it was impossible to investigate the reproductive outcomes of high-grade mosaic embryos because it would be unethical to do so given the disproportionate impact on women, who would most likely be aggrieved by the transfer of fully aneuploid embryos (e.g., by miscarriage). However, this lack of information is not expected to

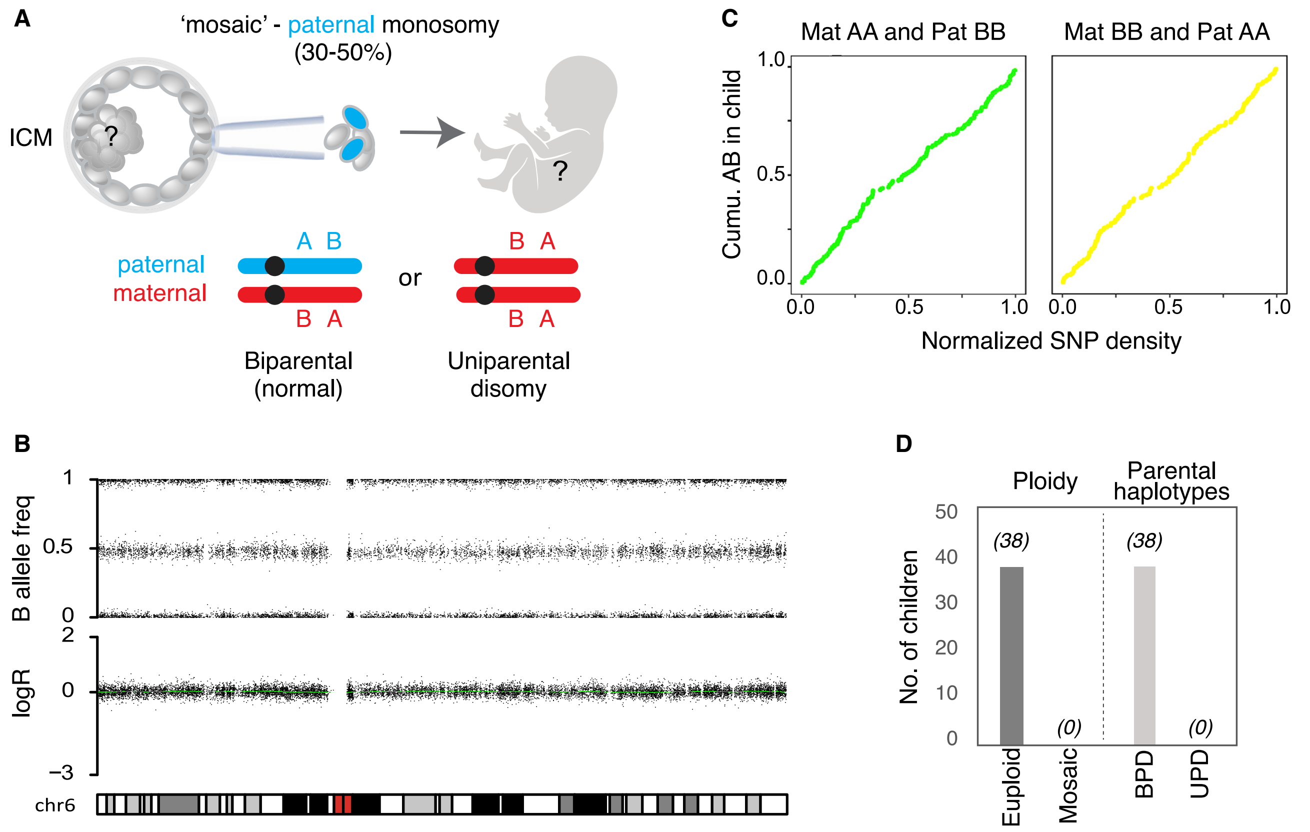

Figure 3. Euploid biparental inheritance in children born from "mosaic" embryo transfer
(A) Illustration of a mosaic paternal monosomy inferred from the trophectoderm biopsy. The fetal tissues derive from the inner cell mass, which
might contain biparental or uniparental chromosomes or a mixture of them. Supporting SNPs where the maternal and paternal genotypes are homozygous but carry opposite alleles (AA
and BB or vice versa) can be used for determining the presence or absence of parental chromosomes.
(B) LogR and B allele frequencies for chromosome 6 from a child born from group C.
(C) Cumulative AB genotypes in the child of supporting SNPs across chromosome 6.
(D) Number (No.) of children investigated with post
natal SNPa testing. Total number of samples showing euploid or mosaic karyotype ("ploidy") or containing both parental chromosomes (biparental disomy, BPD) or two homologous chromosomes from the same parent (UPD).

significantly impact the effectiveness of PGT-A cycles because high-grade mosaicism was detected in only 1.6% of embryos analyzed (Figure 1A). It should be noted that the results reported in this study were obtained through the analysis of raw NGS data independent from any proprietary diagnostic algorithm or chromosome-specific consideration commonly used by PGT-A laboratories. Accordingly, our approach provides all PGT laboratories with common ground that is highly reproducible and independent of specific individual settings. Nevertheless, it is important that each laboratory tests and validates its specific algorithms in prospective non-selection studies like the one presented here. Furthermore, these findings concern whole-chromosome mosaic aneuploidies only and cannot be extended to segmental mosaic configuration, which might follow different trajectories. Using a similar embryo-disaggregation design, we have recently demonstrated that most segmental unbalances detected in human blastocysts are indeed in a mosaic state, in opposition to what is observed for whole-chromosome aneuploidies, which mostly originate after meiotic segregation errors.[1,30] Future non-selection studies are needed for the investigation of the clinical predictive values of mosaic segmental abnormalities detected in TE biopsies.

Although it remains unclear whether aneuploid cells arrest, become senescent or apoptotic, or are precursors of placental mosaicism,[31] our data also support the existence of early developmental bottleneck events able to normalize mosaicism-initiating post-zygotic aneuploidies in human embryos. As shown here, low- and medium-grade mosaic embryos have similar reproductive potential as uniformly euploid embryos and, according to our modeling, deselecting them from clinical treatment[10] would have resulted in a reduction of LBRs of up to 36% in IVF/PGT-A cycles. Considering that in the US alone, more than 50,000 PGT-A cycles are performed every year, the inclusion of putative mosaic embryos in transfer selection would rescue a remarkable number of embryos and live births. In light of this experience, we encourage the preliminary use of data from non-selection trials before being incorporating new

PGT-A algorithms and aneuploidy classification criteria in routine practice.

Data and code availability

All necessary data are available in the main text or supplemental material. For Figure S3, all data points are provided in the supplemental information. The complete dataset of anonymized clinical records is available upon request.
The code for SNP-array-based UPD and mosaicism analysis of trios is publicly available at the following link: https://github.com/Meiomap/TrioAnalysis.

Supplemental information

Supplemental information can be found online at https://doi.org/10.1016/j.ajhg.2021.11.002.

Acknowledgments

The study was supported by Igenomix. The authors also acknowledge the support of a European Research Council consolidator grant (724718-ReCAP), the Novo Nordisk Foundation Young Investigator Award (NNF15OC0016662), ReproUnion, and the Danish National Research Foundation (center grant 6110-00344B). Furthermore, the authors would like to thank all colleagues and collaborators who have contributed to this study and to acknowledge their work in data collection and treatment monitoring across all the participating sites and institutions. A special acknowledgment is given to the following individuals for their contributions: Carlos Gomez De la Cruz and Emilio de la Fuente Lucena from Igenomix; Cinzia Gentile from Genera Veneto, GeneraLife IVF; Erminia Alviggi from Ruesch Clinic, GeneraLife IVF; Antonella Smeraldi and Amalia Cesana from Humanitas; and Valentina Zicaro from Demetra.

Declaration of interests

A.C., M.P., L.G., C.P., M.F., M.F., J.C., and C.R. are full-time employees of Igenomix.

L.R. is the Scientific Director of GeneraLife IVF. She is editor of reproductive biomedicine online and has been associate editor of human reproduction update. She has been the principal investigator of a study sponsored by Merck KGaA. She has received honoraria and consultation fees from Merck, MSD, Ferring, Ibsa, Cooper Surgical, Cook, Nterilizer, Fujifilm-Irvine Scientific, Medea, and Universal Clinics. She is a partner and shareholder of Global Investment Clinics, Genera Health Care, and Nterilizer and has been affiliated with Flam.

D.C. is a full-time employee of GeneraLife IVF, where he is the science and research manager. He received paid lectures from Fujifilm-Irvine Scientific. He received paid consultations from Merck.

F.B. reports personal fees from Fujifilm-Irvine Scientific, outside the submitted work.

A.V. reports personal fees from Gedeon Richter, personal fees from Merck, and personal fees from MSD, outside the submitted work.

E.H. and I.V. are supported by an ERC consolidator grant (724718-ReCAP), the Novo Nordisk Foundation Young Investigator Award (NNF15OC0016662), ReproUnion, and the Danish National Research Foundation (center grant 6110-00344B). The views expressed in this article are those of the authors and not necessarily those of the sponsors. The funders of the study had no role in study design, data collection, data analysis, data interpretation, or writing of this article. The corresponding authors had full access to all the data in the study and had final responsibility for the decision to submit for publication.

E.H. is the executive board member and chair of the scientific advisory board of ReproUnion, which is co-funded by Ferring Pharmaceuticals.

F.M.U. is the scientific director of GeneraLife IVF. He is the president of the Italian Society of Fertility and Sterility (SIFES). He has been the principal investigator of a study sponsored by Merck and by S&R Farmaceutici. He has received honoraria and consultation fees from Merck, Merck Sharp and Dohnme Corporation, Ferring, Institut Biochimique, Cooper Surgical, Cook, Nterilizer, Fujifilm-Irvine Scientific, Medea, and Universal Clinics. He is partner/shareholder of Global Investment Clinics, Genera Health Care, and Nterilizer, and has been of Flam.

C.S. is the head of the scientific advisory board of Igenomix.

The other authors declare no competing interests.

Received: September 15, 2021
Accepted: October 29, 2021
Published: November 18, 2021

References

1. Gruhn, J.R., Zielinska, A.P., Shukla, V., Blanshard, R., Capalbo, A., Cimadomo, D., Nikiforov, D., Chan, A.C.H., Newnham, L.J., Vogel, I., et al. (2019). Chromosome errors in human eggs shape natural fertility over reproductive life span. Science *365*, 1466–1469.
2. Popovic, M., Dhaenens, L., Boel, A., Menten, B., and Heindryckx, B. (2020). Chromosomal mosaicism in human blastocysts: the ultimate diagnostic dilemma. Hum. Reprod. Update *26*, 313–334.
3. van Echten-Arends, J., Mastenbroek, S., Sikkema-Raddatz, B., Korevaar, J.C., Heineman, M.J., van der Veen, F., and Repping, S. (2011). Chromosomal mosaicism in human preimplantation embryos: a systematic review. Hum. Reprod. Update *17*, 620–627.
4. van der Meij, K.R.M., Sistermans, E.A., Macville, M.V.E., Stevens, S.J.C., Bax, C.J., Bekker, M.N., Bilardo, C.M., Boon, E.M.J., Boter, M., Diderich, K.E.M., et al.; Dutch NIPT Consortium (2019). TRIDENT-2: National Implementation of Genome-wide Non-invasive Prenatal Testing as a First-Tier Screening Test in the Netherlands. Am. J. Hum. Genet. *105*, 1091–1101.
5. Van Den Bogaert, K., Lannoo, L., Brison, N., Gatinois, V., Baetens, M., Blaumeiser, B., Boemer, F., Bourlard, L., Bours, V., De Leener, A., et al. (2021). Outcome of publicly funded nationwide first-tier noninvasive prenatal screening. Genet. Med. *23*, 1137–1142.
6. Hsu, L.Y.F., Yu, M.T., Richkind, K.E., Van Dyke, D.L., Crandall, B.F., Saxe, D.F., Khodr, G.S., Mennuti, M., Stetten, G., Miller, W.A., and Priest, J.H. (1996). Incidence and significance of chromosome mosaicism involving an autosomal structural abnormality diagnosed prenatally through amniocentesis: a collaborative study. Prenat. Diagn. *16*, 1–28.
7. Singla, S., Iwamoto-Stohl, L.K., Zhu, M., and Zernicka-Goetz, M. (2020). Autophagy-mediated apoptosis eliminates

aneuploid cells in a mouse model of chromosome mosaicism. Nat. Commun. *11*, 2958.

8. Bolton, H., Graham, S.J.L., Van der Aa, N., Kumar, P., Theunis, K., Fernandez Gallardo, E., Voet, T., and Zernicka-Goetz, M. (2016). Mouse model of chromosome mosaicism reveals lineage-specific depletion of aneuploid cells and normal developmental potential. Nat. Commun. *7*, 11165.
9. Capalbo, A., Hoffmann, E.R., Cimadomo, D., Ubaldi, F.M., and Rienzi, L. (2017). Human female meiosis revised: new insights into the mechanisms of chromosome segregation and aneuploidies from advanced genomics and time-lapse imaging. Hum. Reprod. Update *23*, 706–722.
10. Munné, S., Blazek, J., Large, M., Martinez-Ortiz, P.A., Nisson, H., Liu, E., Tarozzi, N., Borini, A., Becker, A., Zhang, J., et al. (2017). Detailed investigation into the cytogenetic constitution and pregnancy outcome of replacing mosaic blastocysts detected with the use of high-resolution next-generation sequencing. Fertil. Steril. *108*, 62–71.e8.
11. Cram, D.S., Leigh, D., Handyside, A., Rechitsky, L., Xu, K., Harton, G., Grifo, J., Rubio, C., Fragouli, E., Kahraman, S., et al. (2019). PGDIS position statement on the transfer of mosaic embryos 2019. Reprod. Biomed. Online *39* (*Suppl 1*), e1–e4.
12. Practice Committee and Genetic Counseling Professional Group (GCPG) of the American Society for Reproductive Medicine. Electronic address: asrm@asrm.org (2020). Clinical management of mosaic results from preimplantation genetic testing for aneuploidy (PGT-A) of blastocysts: a committee opinion. Fertil. Steril. *114*, 246–254.
13. Zamani Esteki, M., Viltrop, T., Tšuiko, O., Tiirats, A., Koel, M., Nõukas, M., Žilina, O., Teearu, K., Marjonen, H., Kahila, H., et al. (2019). In vitro fertilization does not increase the incidence of de novo copy number alterations in fetal and placental lineages. Nat. Med. *25*, 1699–1705.
14. Huang, A., Adusumalli, J., Patel, S., Liem, J., Williams, J., 3rd, and Pisarska, M.D. (2009). Prevalence of chromosomal mosaicism in pregnancies from couples with infertility. Fertil. Steril. *91*, 2355–2360.
15. Kalousek, D.K., and Dill, F.J. (1983). Chromosomal mosaicism confined to the placenta in human conceptions. Science *221*, 665–667.
16. Greco, E., Minasi, M.G., and Fiorentino, F. (2015). Healthy Babies after Intrauterine Transfer of Mosaic Aneuploid Blastocysts. N. Engl. J. Med. *373*, 2089–2090.
17. Viotti, M., Victor, A.R., Barnes, F.L., Zouves, C.G., Besser, A.G., Grifo, J.A., Cheng, E.H., Lee, M.S., Horcajadas, J.A., Corti, L., et al. (2021). Using outcome data from one thousand mosaic embryo transfers to formulate an embryo ranking system for clinical use. Fertil. Steril. *115*, 1212–1224.
18. Capalbo, A., Wright, G., Elliott, T., Ubaldi, F.M., Rienzi, L., and Nagy, Z.P. (2013). FISH reanalysis of inner cell mass and trophectoderm samples of previously array-CGH screened blastocysts shows high accuracy of diagnosis and no major diagnostic impact of mosaicism at the blastocyst stage. Hum. Reprod. *28*, 2298–2307.
19. Capalbo, A., Rienzi, L., Cimadomo, D., Maggiulli, R., Elliott, T., Wright, G., Nagy, Z.P., and Ubaldi, F.M. (2014). Correlation between standard blastocyst morphology, euploidy and implantation: an observational study in two centers involving 956 screened blastocysts. Hum. Reprod. *29*, 1173–1181.
20. Cimadomo, D., Soscia, D., Vaiarelli, A., Maggiulli, R., Capalbo, A., Ubaldi, F.M., and Rienzi, L. (2019). Looking past the appearance: a comprehensive description of the clinical contribution of poor-quality blastocysts to increase live birth rates during cycles with aneuploidy testing. Hum. Reprod. *34*, 1206–1214.
21. Fischer, C., and Scott, R.T., Jr. (2020). Three simple metrics to define in vitro fertilization success rates. Fertil. Steril. *114*, 6–8.
22. Zegers-Hochschild, F., Adamson, G.D., Dyer, S., Racowsky, C., de Mouzon, J., Sokol, R., Rienzi, L., Sunde, A., Schmidt, L., Cooke, I.D., et al. (2017). The International Glossary on Infertility and Fertility Care, 2017. Fertil. Steril. *108*, 393–406.
23. Lensen, S., Osavlyuk, D., Armstrong, S., Stadelmann, C., Hennes, A., Napier, E., Wilkinson, J., Sadler, L., Gupta, D., Strandell, A., et al. (2019). A Rrndomized trial of endometrial scratching before in vitro fertilization. N. Engl. J. Med. *380*, 325–334.
24. Shi, Y., Sun, Y., Hao, C., Zhang, H., Wei, D., Zhang, Y., Zhu, Y., Deng, X., Qi, X., Li, H., et al. (2018). Transfer of fresh versus frozen embryos in ovulatory women. N. Engl. J. Med. *378*, 126–136.
25. García-Pascual, C.M., Navarro-Sánchez, L., Navarro, R., Martínez, L., Jiménez, J., Rodrigo, L., Simón, C., and Rubio, C. (2020). Optimized NGS approach for detection of aneuploidies and mosaicism in PGT-A and imbalances in PGT-SR. Genes (Basel) *11*, 724.
26. Weksberg, R., Shuman, C., and Beckwith, J.B. (2010). Beckwith-Wiedemann syndrome. Eur. J. Hum. Genet. *18*, 8–14.
27. Kahraman, S., Cetinkaya, M., Yuksel, B., Yesil, M., and Pirkevi Cetinkaya, C. (2020). The birth of a baby with mosaicism resulting from a known mosaic embryo transfer: a case report. Hum. Reprod. *35*, 727–733.
28. Besser, A.G., and Mounts, E.L. (2017). Counselling considerations for chromosomal mosaicism detected by preimplantation genetic screening. Reprod. Biomed. Online *34*, 369–374.
29. Mounts, E.L., and Besser, A.G. (2021). Lack of evidence to support recommendation for prenatal uniparental disomy (UPD) analysis following mosaic embryo transfer. Genet. Med. *23*, 230–231.
30. Girardi, L., Serdarogullari, M., Patassini, C., Poli, M., Fabiani, M., Caroselli, S., Coban, O., Findikli, N., Boynukalin, F.K., Bahceci, M., et al. (2020). Incidence, Origin, and Predictive Model for the Detection and Clinical Management of Segmental Aneuploidies in Human Embryos. Am. J. Hum. Genet. *106*, 525–534.
31. Coorens, T.H.H., Oliver, T.R.W., Sanghvi, R., Sovio, U., Cook, E., Vento-Tormo, R., Haniffa, M., Young, M.D., Rahbari, R., Sebire, N., et al. (2021). Inherent mosaicism and extensive mutation of human placentas. Nature *592*, 80–85.

The American Journal of Human Genetics, Volume *108*

Supplemental information

Mosaic human preimplantation embryos and their developmental potential in a prospective, non-selection clinical trial

Antonio Capalbo, Maurizio Poli, Laura Rienzi, Laura Girardi, Cristina Patassini, Marco Fabiani, Danilo Cimadomo, Francesca Benini, Alessio Farcomeni, Juliana Cuzzi, Carmen Rubio, Elena Albani, Laura Sacchi, Alberto Vaiarelli, Matteo Figliuzzi, Necati Findikli, Onder Coban, Fazilet K. Boynukalin, Ivan Vogel, Eva Hoffmann, Claudia Livi, Paolo E. Levi-Setti, Filippo M. Ubaldi, and Carlos Simón

SUPPLEMENTAL INFORMATION

SUPPLEMENTAL FIGURES

Figure S1 - Chromosome representation of transferred mosaic embryos. Distribution of mosaic chromosomes in embryos from group B (top) and from group C (bottom). Mosaic chromosomes were classified according to mosaicism rate: Low Mosaic Trisomy (20% < mosaicism rate ≤30%), Low Mosaic Monosomy (-30%< mosaic rate ≤-20%), Medium Mosaic Trisomy (30%< mosaic rate ≤50%), Medium Mosaic Monosomy (-50%< mosaic rate ≤-30%).

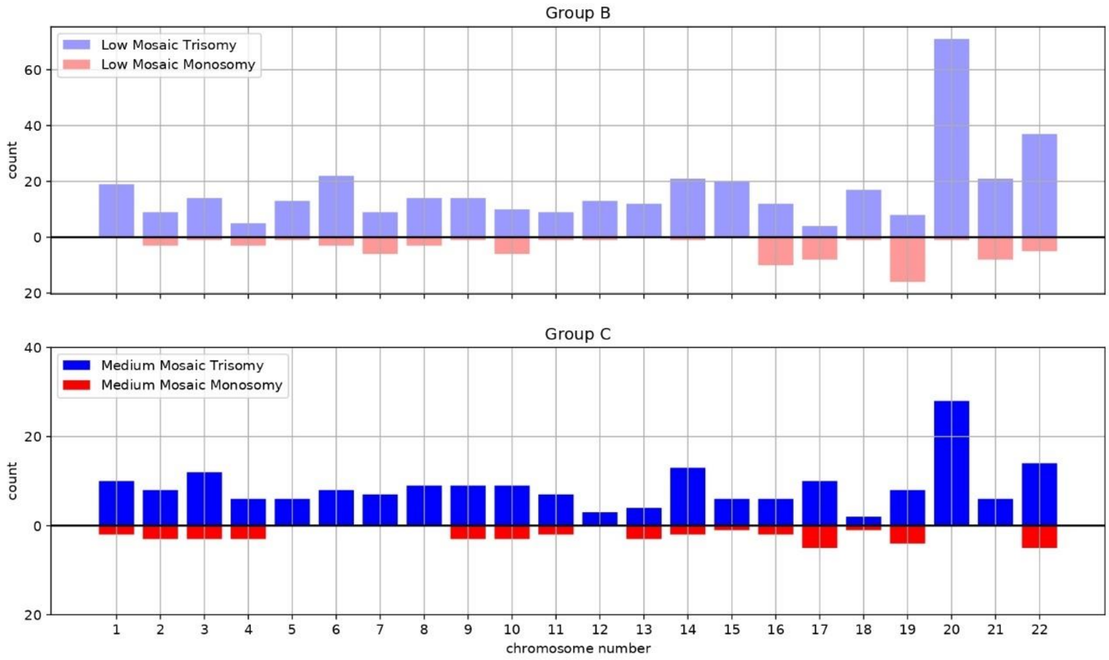

Figure S2 – Euploidy with biparental inheritance in children born from 'mosaic' embryo transfer – full genetic characterization. A) Cumulative AB heterozygous SNPs in the child, where the parents were of genotypes AA and BB (AABBAB- upper row) or BB and AA (BBAAAB – lower row) for all chromosomes. **B)** LogR and B allele frequencies for all chromosomes from a child born from Group C. The green line is the smoothed logR curve. For both panels, chromosome 6 is shown in **Figure 3**.

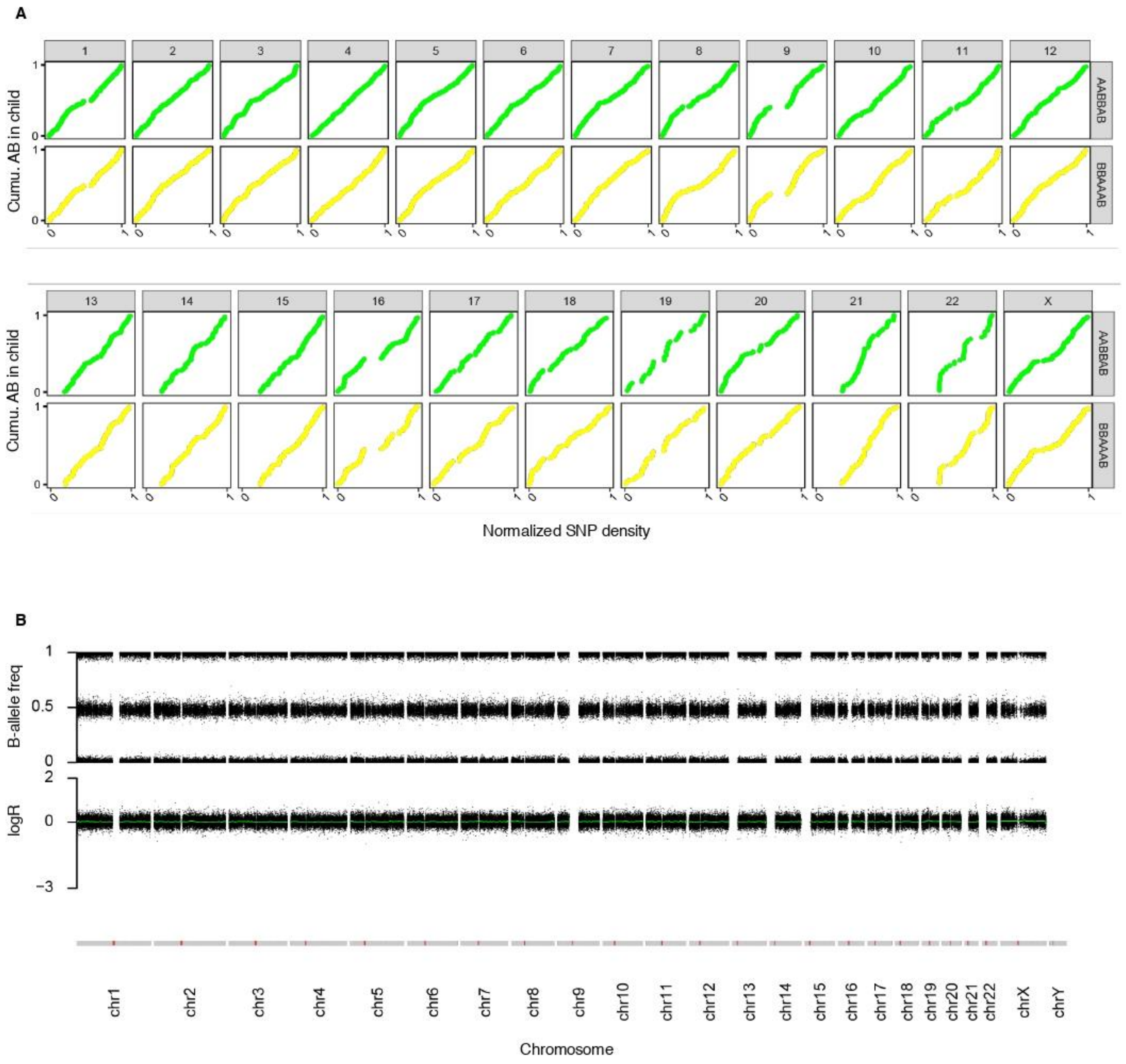

Figure S3 – Estimated impact on cumulative live birth rates in case putative mosaicism embryos are excluded from clinical use. Expected cumulative Live Birth Rate (LBR) per cycle with utilization of all embryos (euploid + putative mosaics) obtained per each cycle is shown in *green* line across the board of female age. *Brown* line projects the situation where putative moderate mosaic embryos are not utilized (- 7% for the observed model and -11% overall relative reduction for the projected model). *Orange* line depicts a scenario where all putative mosaics above 20% variability were excluded from transfer (-24% for the observed model and -36% overall relative reduction for the projected model). **A)** Observed relative reduction in cumulative LBR per cycle based on actual data from this trial. **B)** Optimistic model accounting for the combined probability of LBR in the case all transferable embryos are utilized. This modelling is based on the optimistic scenario, assuming that couples with embryos available for transfer who had not already returned for a subsequent replacement cycles would have the same chance of a pregnancy resulting in a live birth as the recorded LBR per embryo transfer in the whole euploid category (i.e., 43%). The mosaicism incidence is plotted based on the rate observed in the trial.

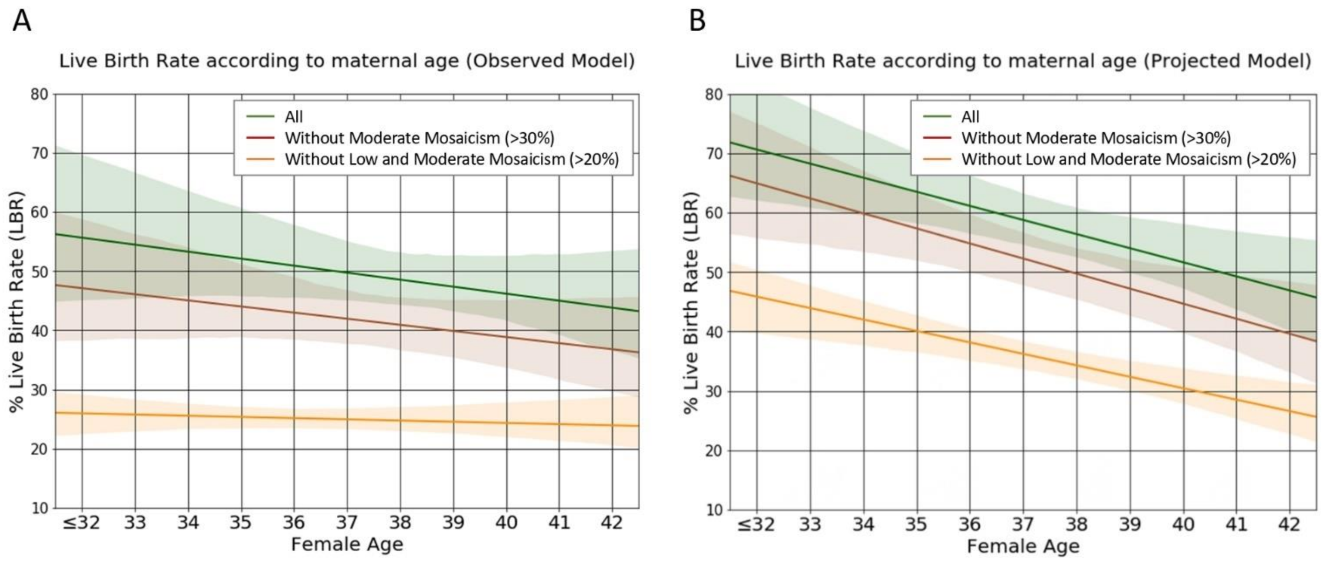

SUPPLEMENTAL TABLES

Table S1 – Demographic data of couples enrolled in the trial.

**DEMOGRAPHIC DATA OF ENROLLED COUPLES**	
Participants, n	783
Embryo transfer procedures, n	897
Mean female age (SD)	37.50 (+ 3.3)
BMI female	21.7 (+ 2.7)
FSH (mIU/mL), mean (+ SD)	8.0 (+ 4.2)
AMH (ng/mL), mean (+ SD)	2.8 (+ 2.9)
**Indication to PGT-A per cycle**	
Advanced Maternal Age (AMA), n (%)	576/783 (73.6%)
Repeated Implantation Failures (RIF), n (%)	32/783 (4.1%)
Repeated Pregnancy Loss (RPL), n (%)	28/783 (3.6%)
AMA + RIF, n (%)	27/783 (3.4%)
AMA + RPL, n (%)	13/783 (1.7%)
No Indication, n (%)	107/783 (13.7%)
**Protocol per cycle**	
Antagonist, n (%)	706/783 (90.2%)
Antagonist, n (%)	16/783 (2.0%)
DuoStim, n (%)	61/783 (7.8%)
**Semen**	
Ejaculated, n (%)	768/783 (98.1%)
Surgical, n (%)	14/783 (1.8%)
Donated, n (%)	1/783 (0.1%)
Sperm concentration [millions/ml], mean (+ SD)	32.8 (+ 26.1)
Sperm progressive motility [A+B%], mean (+ SD)	38.7 (+ 17.1)
Sperm morphology [% sperm with normal morphology], mean (+ SD)	4.4 (+ 2.6)
**Cycle data**	
Retrieved oocyte, mean (+ SD)	9.1 (+ 5.0)
2pn zygotes, mean (+ SD)	6.7 (+ 3.6)
Biopsied embryo [n], (mean + SD)	2,874 (3.4 + 1.9)
Euploid embryos, n (%)	1,774/2,874 (61.7%)
EUPLOID (<20%), n (%)	941 /2,874 (32.7%)
EUPLOID (20%-30%), n (%)	541/2,874 (18.8%)
EUPLOID (30%-50%), n (%)	292/2,874 (10.2%)
Aneuploid embryos (>50%), n (%)	1,100/2,874 (38.3%)

Table S2 – Additional mosaicism metrics and their association with single embryo transfer outcomes.

	Biochem Pregnancy Loss		Miscarriage		Live birth	
	YES	NO	YES	NO	YES	NO
Mean mosaicism rate<br>% (95%CI)	20.4%<br>(19.6-21.3)	22.6%<br>(20.3-24.9)	20.4%<br>(19.5-21.2)	20.8%<br>(18.1-23.5)	20.9%<br>(20.1-21.7)	20.4%<br>(19.6-21.3)
Mean number of mosaic chromosomes<br>(95%CI)	0.98<br>(0.59-1.37)	0.98<br>(0.83-1.14)	1.1<br>(0.54-1.68)	0.97<br>(0.80-1.13)	0.97<br>(0.81-1.13)	1.0<br>(0.86-1.15)
Incidence of complex mosaic<br>(>3 chr)<br>% (n) P-value (Y Vs N)	12.1%<br>(7/58)	13.4%<br>(59/440)<br>P=0.5	11.5%<br>(6/52)	13.7%<br>(53/388)<br>P=0.82	13.7%<br>53/386	12.5%<br>64/511<br>P=0.6
Incidence of complex mosaic<br>(>5 chr)<br>% (n) P-value (Y Vs N)	3.4%<br>(2/58)	5.2%<br>(23/440)<br>P=0.5	7.7% (4/52)	4.9%<br>(19/388)<br>P=0.33	4.9%<br>(19/386)	5.5%<br>(28/511)<br>P=0.8

Table S3 – Chromosome-specific analysis on the primary outcome measure. Projected and observed cumulative birth rates in cases where i) all embryos with NGS profiles with chromosomal copy number variations below 50% are transferred, ii) if embryos with >30% variation are not transferred and iii) if embryos with >20% variation are not transferred.

Chromosome involved in mosaicism		LBR .00	LBR 1.00	Total
1	Count	21	17	38
	% within chr_mosa_MAX	55.3%	44.7%	100.0%
	% within LBR	4.1%	4.4%	4.2%
2	Count	16	12	28
	% within chr_mosa_MAX	57.1%	42.9%	100.0%
	% within LBR	3.1%	3.1%	3.1%
3	Count	18	14	32
	% within chr_mosa_MAX	56.3%	43.8%	100.0%
	% within LBR	3.5%	3.6%	3.6%
4	Count	10	4	14
	% within chr_mosa_MAX	71.4%	28.6%	100.0%
	% within LBR	2.0%	1.0%	1.6%
5	Count	11	11	22
	% within chr_mosa_MAX	50.0%	50.0%	100.0%
	% within LBR	2.2%	2.8%	2.5%
6	Count	27	25	52
	% within chr_mosa_MAX	51.9%	48.1%	100.0%
	% within LBR	5.3%	6.5%	5.8%
7	Count	10	6	16
	% within chr_mosa_MAX	62.5%	37.5%	100.0%
	% within LBR	2.0%	1.6%	1.8%
8	Count	20	12	32
	% within chr_mosa_MAX	62.5%	37.5%	100.0%
	% within LBR	3.9%	3.1%	3.6%
9	Count	17	16	33
	% within chr_mosa_MAX	51.5%	48.5%	100.0%
	% within LBR	3.3%	4.1%	3.7%
10	Count	18	15	33
	% within chr_mosa_MAX	54.5%	45.5%	100.0%
	% within LBR	3.5%	3.9%	3.7%
11	Count	8	4	12
	% within chr_mosa_MAX	66.7%	33.3%	100.0%
	% within LBR	1.6%	1.0%	1.3%
12	Count	11	10	21
	% within chr_mosa_MAX	52.4%	47.6%	100.0%
	% within LBR	2.2%	2.6%	2.3%
13	Count	16	10	26
	% within chr_mosa_MAX	61.5%	38.5%	100.0%
	% within LBR	3.1%	2.6%	2.9%
14	Count	30	22	52
	% within chr_mosa_MAX	57.7%	42.3%	100.0%
	% within LBR	5.9%	5.7%	5.8%
15	Count	18	13	31
	% within chr_mosa_MAX	58.1%	41.9%	100.0%
	% within LBR	3.5%	3.4%	3.5%
16	Count	21	17	38
	% within chr_mosa_MAX	55.3%	44.7%	100.0%
	% within LBR	4.1%	4.4%	4.2%
17	Count	13	13	26
	% within chr_mosa_MAX	50.0%	50.0%	100.0%
	% within LBR	2.5%	3.4%	2.9%
18	Count	20	11	31
	% within chr_mosa_MAX	64.5%	35.5%	100.0%
	% within LBR	3.9%	2.8%	3.5%
19	Count	41	24	65
	% within chr_mosa_MAX	63.1%	36.9%	100.0%
	% within LBR	8.0%	6.2%	7.2%
20	Count	72	71	143
	% within chr_mosa_MAX	50.3%	49.7%	100.0%
	% within LBR	14.1%	18.4%	15.9%
21	Count	36	25	61
	% within chr_mosa_MAX	59.0%	41.0%	100.0%
	% within LBR	7.0%	6.5%	6.8%
22	Count	57	34	91
	% within chr_mosa_MAX	62.6%	37.4%	100.0%
	% within LBR	11.2%	8.8%	10.1%
Total	Count	511	386	897
	% within chr_mosa_MAX	57.0%	43.0%	100.0%
	% within LBR	100.0%	100.0%	100.0%

Table S4: Cumulative live birth rate per cycle data points according to the observed and projected model.

Female Age	Live Birth Rate according to maternal age (Observed Model)						Live Birth Rate according to maternal age (Projected Model)					
	All		Without moderate mosaicism (>30%)		Without low+ mod mosaicism (>20%)		All		Without moderate mosaicism (>30%)		Without low+mod mosaicism (>20%)	
	% (n)	95% CI	% (n)	95% CI	% (n)	95% CI	% (n)	95% CI	% (n)	95% CI	% (n)	95% CI
≤ 32	43.5 (27/62)	31.2-55.9	40.3 (25/62)	28.1-52.5	25.8 (16/62)	14.9-36.7	63.3 (39.3/62)	51.3-75.3	60.9 (37.8/62)	48.8-73.1	46.5 (28.8/62)	34.1-58.9
33	68 (17/25)	49.7-86.3	52 (13/25)	32.4-71.6	28 (7/25)	10.4-45.6	77.4 (19.3/25)	61.0-93.8	69.8 (17.4/25)	51.8-87.8	48.1 (12/25)	28.5-67.7
34	43.8 (21/48)	29.7-57.8	33.3 (16/48)	20.0-46.7	20.8 (10/48)	9.3-32.3	54.8 (26.3/48)	40.8-68.9	45.3 (21.8/48)	31.2-59.4	33.4 (16.1/48)	20.1-46.8
35	60 (39/65)	48.1-71.9	53.8 (35/65)	41.7-66.0	26.2 (17/65)	15.5-36.8	69.9 (45.4/65)	58.7-81.0	63.1 (41/65)	51.4-74.8	40.1 (26.1/65)	28.2-52.0
36	47.6 (59/124)	38.8-56.4	41.9 (52/124)	33.3-50.6	27.4 (34/124)	19.6-35.3	59.8 (74.2/124)	51.2-68.5	55.2 (68.4/124)	46.4-63.9	39.6 (49.2/124)	31.0-48.2
37	57.8 (48/83)	47.2-68.5	49.4 (41/83)	38.6-60.2	25.3 (21/83)	15.9-34.7	67.2 (55.8/83)	57.1-77.3	60.3 (50.1/83)	49.8-70.9	39.9 (33.1/83)	29.3-50.4
38	45.9 (50/109)	36.5-55.2	40.4 (44/109)	31.2-49.6	23.9 (26/109)	15.9-31.9	53.9 (58.7/109)	44.5-63.2	48.4 (52.7/109)	39.0-57.8	32.4 (35.3/109)	23.6-41.2
39	51.6 (49/95)	41.5-61.6	43.2 (41/95)	33.2-53.1	25.3 (24/95)	16.5-34.0	59.3 (56.4/95)	49.4-69.2	51.8 (49.2/95)	41.8-61.9	34.7 (32.9/95)	25.1-44.2
40	42.4 (36/85)	31.8-52.9	36.5 (31/85)	26.2-46.7	21.2 (18/85)	12.5-29.9	46.7 (39.7/85)	36.1-57.3	41.9 (35.6/85)	31.4-52.4	27.3 (23.2/85)	17.8-36.8
41	51.4 (36/70)	39.7-63.1	44.3 (31/70)	32.6-55.9	30 (21/70)	19.3-40.7	55.2 (38.6/70)	43.5-66.8	48.3 (33.8/70)	36.6-60.0	33.4 (23.4/70)	22.4-44.5
42	35.3 (12/34)	19.2-51.4	26.5 (9/34)	11.6-41.3	20.6 (7/34)	7.0-34.2	39.1 (13.3/34)	22.7-55.5	30.3 (10.3/34)	14.8-45.7	23.1 (7.9/34)	8.9-37.3

SUPPLEMENTAL METHODS: CLINICAL TRIAL REPORTING

Participant's inclusion/exclusion criteria and clinical trial flow-chart

Couples were included in the trial if below the age of 45 (female partner), using their own oocytes, undergoing ICSI for all oocytes and had at least one transferrable embryo available (euploid or low/medium-grade mosaic). Women with a history of unilateral oophorectomy, recurrent spontaneous abortion, diagnosis of the polycystic ovary syndrome, or uterine abnormality (e.g., Müllerian duct anomaly, adenomyosis, submucous myoma, intra uterine adhesion, or scarred uterus) were excluded. Women were also excluded if they had a chronic medical condition that has been associated with adverse pregnancy outcomes (i.e., endometriosis, autoimmune disorders such as severe systemic lupus erythematosus). All the couples were screened with the use of standard karyotyping, and those with numerical or structural uniform or mosaic abnormalities were excluded from the trial. Individuals showing unstable illnesses or medical conditions that may put their safety at risk were also excluded from the study (i.e., cancer, severe obesity). Cases where the only embryo available was of the worst morphological grade (according to the Gardner's criteria) were also excluded from the study. This criterion was introduced to mitigate an intrinsic bias, as euploid blastocysts of very poor morphological grade were shown to result in lower live birth and higher miscarriage rate compared to embryos with better morphology[1].

A total of 1,603 IVF cycles from 1,190 couples were assessed for eligibility from September 2018 through December 2019. Of these cycles, 266 were excluded from the study as 41 didn't produce any normally fertilised zygotes (41/1,603; 2.6%) and 225 produced blastocysts ineligible for biopsy (225/1,603; 14%). In addition, of the 1,337 cases undergoing trophectoderm biopsy and PGT-A, 490 were excluded as they led to all aneuploid embryos (490/1,337; 36.6%). Finally, a total of 783 couples were enrolled in this trial, 41 of which underwent two stimulation cycles. Overall, 824 stimulations led to 897 single-embryo transfers, with 50 couples receiving two SET and 7 couples receiving three SET. All remaining 676 couples received one SET at the time the enrolment to this trial was closed. Embryo morphology-based embryo selection led to the transfer of 484 uniform euploid embryos (Group A), 282 putative low mosaic embryos (Group B) and 131 putative moderate mosaic embryos (Group C) (**Figure 2**). Baseline characteristics and main IVF cycle outcomes of couples that entered the study are shown in **Table S1**.

Primary and secondary outcomes could be monitored for all cases, apart from mean gestational age at birth and mean birth weight which were obtained in 97% of cases. A minority of miscarriages could be characterized cytogenetically by product of conception (POC) analysis (n=4/52; 7.7%) and only 26 pregnancies underwent prenatal diagnosis

(PND;n=26/388, 6.7%). A total of 50 samples were collected from either putative mosaic (n=36) and uniformly euploid (n= 14) embryo-derived newborns. Of these, 38 passed QC and were selected for molecular testing follow-up involving postnatal karyotyping and genotyping. All remaining cases from putative mosaic embryos declined to participate in this phase of the study.

Description of primary and secondary outcome measures

The primary outcome measure was sustained implantation rate[3], defined as live birth rate (LBR) per transferred embryo according to the WHO and ICMART International Glossary on Infertility and Fertility Care[2]. The LBR is calculated as the number of newborns delivered on or after 22 weeks of gestation over the number of embryos replaced. In the event of a single-embryo transfer as occurred in this study for all cases, the metric is identical to delivery rate per transfer. The secondary outcome was miscarriage rate defined as the spontaneous loss of an intra-uterine pregnancy prior to 20 completed weeks of gestational age. This included the evaluation of pregnancy rate (PR), biochemical pregnancy (BP), clinical miscarriage (CM). Mean gestational age at birth and birth weight were also collected as additional neonatal outcomes. Adverse outcomes included the detection of chromosomal abnormalities, including uniparental disomy, in the miscarried product of conception (POC), during prenatal diagnosis (PND; amniocentesis/chorionic villi sampling, CVS) and/or at birth.

The implication of excluding putative mosaic embryos from clinical use has been evaluated in consideration of the potential loss of live births in a given IVF treatment cycle (Cumulative LBR, CLBR per cycle) assuming two scenarios: i) using actual data from embryo transfer in the study period excluding live births achieved from low and moderate mosaic embryos; ii) by modelling the optimistic scenario where all transferable embryos are replaced[4,5].

Outcome measures were described according to the International Glossary on Infertility and Fertility Care[2] and standard definitions:

Pregnancy rate (PR): The number of couples with positive serum level of β-human chorionic gonadotropin (βhCG) ($\geq$ 25 mIU/mL) per embryo transfer.

Live birth rate (LBR): The number of deliveries that resulted in at least one live birth per embryo transfer. Live birth is defined as the complete expulsion or extraction from a woman of a product of conception after 22 weeks of gestation, which, after such separation, breathes or shows any other evidence of life, such as heartbeat, umbilical cord pulsation or definite movement of voluntary muscles, irrespective of whether the umbilical cord has been cut or the

placenta is attached. In our study all embryo transfer procedures were of Single Embryo Transfer. Accordingly, LBR can be approximated as the number of live births divided by the number of SET procedures.

Implantation rate (IR): The number of gestational sacs observed by vaginal ultrasound at the 5th gestational week divided by the number of embryos transferred.

Clinical miscarriage rate (CMR)*:* Number of spontaneous pregnancy losses before week 20 in which a gestational sac/s was previously observed, per number of pregnancies, excluding ectopic pregnancy.

Biochemical pregnancy rate (BPR): Number of pregnancies diagnosed only by βhCG detection without a gestational sac visualized by vaginal ultrasound at the 5th week of pregnancy, per number of pregnancies.

Ectopic pregnancy rate (EPR): Number of pregnancies outside the uterine cavity, diagnosed by ultrasound, surgical visualization or histopathology, per number of pregnancies. For cumulative outcomes, we considered the clinical results obtained from all the embryo transfers performed in the same arm of the study up to 12 months follow-up.

Cumulative live birth rate (CLBR)*:* The number of deliveries that resulted in at least one live birth (as previously defined), per total number of couples receiving embryo transfer following the same type of transfer arm into which the couple was randomized for up to 12 months follow-up period. All clinical outcomes are presented as percentage (%).

Cumulative live birth rate per cycle (CLBR per cycle): the number of deliveries that resulted in at least one live birth (as previously defined) in a single ovarian stimulation cycle.

IVF treatment and embryology procedures

Standard IVF procedures were carried out for enrolled couples without any specific intervention apart from those specified. Ovarian stimulation and embryo culture and transfer were performed according to standard practice at each clinic. In all centres, embryo biopsy procedures were performed according to a previously published protocol[6]. Briefly, expanded blastocysts with or without herniating cells were submitted to TE biopsy. The embryo was anchored to the holding pipette maintaining the ICM at 7 o’clock orientation, allowing the biopsy to be performed at 2 o’clock and minimise interference with the ICM. A diode laser was employed to facilitate the opening of the zona pellucida and allow the passage of the biopsy pipette. Media was gently blown on the TE layer to detach the cells from the zona pellucida. Once detached, 3-10 cells were gently aspirated into the pipette and laser pulses were

directed to the junctions connecting the cells whilst moderate aspiration was applied. Once fully detached, the biopsied cells were released in the same drop, next to the embryo.

Tubing of biopsied cells was performed by briefly washing the specimen in PBS-based solution and transferring it into a 0.2mL sterile PCR tube with a final volume of <2μL.

The tubes were maintained at 4˚C (at the laboratory and during shipment) until arrival at the Igenomix Italy laboratory where they were processed and analysed. Biopsied embryos were cryopreserved using standard vitrification protocol employed by the specific clinic[7]. Euploid embryos were subsequently warmed using the standard warming protocol employed by the specific clinic and transferred to couples either in natural or stimulated embryo transfer cycles arranged as per routine in the specific clinic.

NGS analysis of TE biopsy samples

PGT-A analysis was performed using a semiautomated Next-generation sequencing (NGS) protocol (Ion Reproseq PGS Kit, Thermo Fisher Scientific, MA, USA) with Ion Chef™ equipment for library preparation and multiplexing up to 24-96 samples on a S5 XL sequencer (520 and 530 chips respectively). For individual samples, the most important QC parameters were i) the number of reads (required to be > 70,000 for PGT-A and > 120,000 for PGT-SR), ii) the dispersion/noise of the profile as measured by the mean absolute percent deviation (MAPD) (required to be < 0.3), and iii) the number of duplicates (required to be < 30%). A sample was considered informative if all these parameters were met.

Sequencing data obtained by the S5 sequencer were processed and transferred to Ion Reporter software for analysis (Thermo Fisher Scientific). Chromosome copy number values were calculated per each sample/chromosome and classified in a binary way: euploid (< 50% aneuploid) and aneuploid (≥ 50% aneuploid). Following the completion of all embryo transfer procedures included in the interim analysis, the Euploid category was further elaborated and the NGS data results subdivided in uniformly euploid (<20% aneuploid), low-putative mosaic (20-30%) and moderate putative mosaic (30-50%). This elaboration was independent from any proprietary diagnostic algorithm used in PGT-A laboratories. It primarily consists in the analysis of raw sequencing data with the specific protocol employed here without any chromosome-specific consideration. The NGS protocol used in this study has been extensively validated in our laboratories and shown to be capable of detecting mosaicism in fibroblast cell line

mixture models and diluted genomic DNA samples mimicking mosaicism[8]. Embryo transfer outcomes were then compared across the three different Euploid categories.

Reproducibility of PGT-A procedures and clinical outcomes consistency across the 5 participating centres was previously established[9]. The accuracy of the NGS protocol employed in this study was previously validated for mosaicism detection from cell line mixture models and embryo re-biopsies[8,10].

Additional data analysis and results from the clinical trials

We also evaluated if embryos showing putative mosaic profiles in multiple chromosomes were associated with different transfer outcomes. For this purpose, we assessed all possible metrics associated with chromosome copy number variations and main transfer outcomes (**Table S2**). Mean mosaicism rate (defined as average chromosome copy number value among putative mosaic chromosomes) was not increased in BPL: 20.4% (95%CI=19.6-21.3) vs 22.6% (95%CI=20.3-24.9); embryos resulting in miscarriage vs ongoing pregnancies: 20.4% (95%CI 19.5-21.2) vs 20.8% (95%CI=18.1-23.5), respectively; and for embryos resulting in LB vs embryos failing to implant: 20.9% (95%CI 20.1-21.7) vs 20.4% (95%CI=19.6-21.3), respectively. Mean number of mosaic chromosomes (defined as the average number of distinct putative mosaic chromosomes) was also similar between LBR and control group (**Table S2**).

Also, the absolute number of chromosomal profiles consistent with mosaicism, more than 3 or more than 5 per embryo (commonly defined as complex mosaics) was not associated with BPL (OR=1.00; 95%CI=0.85-1.18), miscarriage (OR=1.05; 95%CI=0.89-1.23) or LBR outcome (OR=0.98; 95%CI=0.91-1.07). Furthermore, we did not detect any putative mosaic chromosome-specific profile associated with LBR outcome (**Table S2**).

Genotyping analysis follow-up strategy of families with babies born following putative mosaic embryo transfer

We contacted all families achieving a live birth following the transfer of a putative mosaic embryo (n= 176). Of these, 36 accepted to enrol in the follow up study, all other families declined their participation to further studies. QC analysis of the collections revealed that 27 (75.0%) met acceptance criteria for all specimens in the trio. As a reference, we selected a random cohort of families that achieved live birth following the transfer of a uniformly euploid embryo. Of the 13 sample trios received, 11 (84.6%) passed QC and could be employed as controls.

Sample collection procedure was performed autonomously by consenting couples using sterile buccal swabs ("FLOQSwabs", Copan diagnostics) provided by Igenomix laboratory.

Buccal cells were collected by firmly pressing and rotating the swab against the inside of the inner cheek for 1 minute, using an up and down motion. The swab was then put back into the original tube, which was labelled with the individual's full name and date of birth. Specimens were sent to Igenomix Italy laboratory at room temperature and, upon arrival, immediately processed for DNA extraction.

SNP Genotyping analysis of trios was conducted double-blind. We genotyped DNA extracted from buccal swabs from both parents and the live born using the Illumina CytoSNP v12 array that were processed on a NextSeq (Illumina Inc.), following the instructions and quality control by the manufacturer. We used GenCall score of 0.75 rather than 0.15 for clinical tests to determine chromosome content using the logR and B allele frequencies. Additionally, to determine parental haplotypes in the child, we selected homozygous SNPs of opposite genotypes in the parents (AA and BB or vice versa, termed supporting SNPs) and plotted the cumulative AB genotype of the child at each SNP. The false discovery rate of the expected AB genotype is less than 0.002% based on the AB genotypes across SNPs where the parental genotypes were the same (e.g., AA and AA for maternal and paternal genotypes, but AB in the child). The code for SNP array based UPD and mosaicism analysis of trios is publicly available at the following link: https://github.com/Meiomap/TrioAnalysis.

Estimated impact on cumulative live birth rates in case low and medium grade mosaicism embryos are excluded from clinical use.

The implication of excluding putative mosaic embryos from clinical use has been evaluated in consideration of the potential loss of live births in a given IVF treatment cycle (CLBR per cycle). The cumulative LBR for a complete cycle is defined as the chance of live birth from an ovarian stimulation cycle including all subsequent FETs from that cycle [3]. Two scenarios were analysed: i) using actual data from embryo transfer in the study period excluding live births achieved from low and moderate mosaic embryos; ii) by modelling the optimistic scenario where all transferable embryos are replaced. For this second approach, a probabilistic projection was computed accounting for all euploid embryos with or without putative mosaic embryos produced from a single ovarian stimulation cycle and considering the combined probability of achieving a live birth based on the available embryos. In this model, the CLBR per cycle was computed by an optimistic approach, that is assuming that all available embryos are transferred

in a given cycle and with a defined probability of success. Live birth rate per euploid or mosaic embryo was the actual value observed in the study across the three study groups (i.e., 43%; **Figure S2**). The observed and projected CLBR per cycle analysis (with and without the clinical use of putative mosaic embryos) is shown across all female ages.

SUPPLEMENATAL REFERENCES

1. Cimadomo, D. et al. Looking past the appearance: a comprehensive description of the clinical contribution of poor-quality blastocysts to increase live birth rates during cycles with aneuploidy testing. Human reproduction (Oxford, England) 34, 1206–1214 (2019).

2. Zegers-Hochschild, F. et al. The International Glossary on Infertility and Fertility Care, 2017. Human reproduction (Oxford, England) 32, 1786–1801 (2017).

3. Fischer, C. & Scott, R. T. Three simple metrics to define in vitro fertilization success rates. Fertility and sterility 114, 6–8 (2020).

4. Smith, A. D. A. C., Tilling, K., Nelson, S. M. & Lawlor, D. A. Live-Birth Rate Associated With Repeat In Vitro Fertilization Treatment Cycles. JAMA 314, 2654–2662 (2015).

5. Malizia, B. A., Hacker, M. R. & Penzias, A. S. Cumulative live-birth rates after in vitro fertilization. The New England journal of medicine 360, 236–43 (2009).

6. Capalbo, A. et al. Correlation between standard blastocyst morphology, euploidy and implantation: an observational study in two centers involving 956 screened blastocysts. Human reproduction (Oxford, England) 29, 1173–81 (2014).

7. Ubaldi, F. M. et al. Reduction of multiple pregnancies in the advanced maternal age population after implementation of an elective single embryo transfer policy coupled with enhanced embryo selection: pre- and post-intervention study. Human reproduction (Oxford, England) 30, 2097–106 (2015).

8. García-Pascual, C. M. et al. Optimized NGS Approach for Detection of Aneuploidies and Mosaicism in PGT-A and Imbalances in PGT-SR. Genes 11, (2020).

9. Capalbo, A. et al. Consistent and reproducible outcomes of blastocyst biopsy and aneuploidy screening across different biopsy practitioners: a multicentre study involving 2586 embryo biopsies. Human reproduction (Oxford, England) 31, 199–208 (2016).

10. Girardi, L. et al. Incidence, Origin, and Predictive Model for the Detection and Clinical Management of Segmental Aneuploidies in Human Embryos. The American Journal of Human Genetics 106, 525–534 (2020).
